# The “exaptation” of linguistic implicit strategies

**DOI:** 10.1186/s40064-016-2788-y

**Published:** 2016-07-18

**Authors:** Edoardo Lombardi Vallauri

**Affiliations:** Università Roma Tre, via Ostiense 236, 00146 Rome, Italy

## Abstract

Implicit strategies are known to increase persuasion performances. Implicits of content (vagueness, implicatures) and implicits of responsibility (presuppositions, topics) will be compared semiotically to non-linguistic implicits such as images and sounds. The results of psycholinguistic and neurolinguistic experiments will be used to propose that presuppositions and topics arose in language as means to spare addressees processing effort on already known contents, but they were subsequently “exapted” to spare effort on unknown marginal contents, and eventually to reduce the probability for doubtful contents to be processed thoroughly and rejected. This will be shown by many examples from commercial advertising and political propaganda.

## What can be implicit in language

It is well known that language use allows for the encoding of information in more or less explicit ways. We will suggest that what can remain implicit in a linguistic message is mainly two kinds of things: its *content* (“Implicitness of content” section) and the *responsibility* of the source in proposing it to the addressee(s) (“Implicitness of responsibility and its “exaptation”” section). We will underline the resemblance between linguistic implicits and other forms of communication (typically, visual and musical), especially as concerns their persuasive effectiveness (“The similarity between implicits and non-linguistic communication: persuasive effectiveness” section). We will propose that the primary function of certain implicits, mainly presuppositions and topics, is to save encoding and decoding effort; but also that they developed the further function of *distracting the addressee’s attention* from the introduction of questionable contents, which ends up in reducing the risk that they are critically challenged and refused (“Presuppositions as reducers of processing effort” section). We will point out that this effect is stronger in public that in private communication (“High diffusion of a message reduces its challengeability” section), and we will support our claims by showing how they are instantiated in typical persuasive texts, such as commercial advertisements and political propaganda (“Some evidence from Italian commercial advertising and political propaganda” section).

## Implicitness of content

The most obvious thing that can remain implicit in a linguistic utterance is some part of its *notional content*. This typically happens when some content is *implicated*, or expressed in a *vague* manner. We will shortly present both cases in the next sections.

### Implicatures

Implicatures are content which is inferrable from the utterance and its context, though not explicitly expressed:

1. a. Are you going to Bengasi next summer?
  b. You know, Lybia is very dangerous, presently…

In (1b), the idea that “we aren’t going to Bengasi next summer” is not explicitly expressed. The addressee can implicate it, starting from shared knowledge such as the fact that (for the Cooperation Principle) the speaker is aware of answering the question in (1a), that Bengasi is in Lybia, that when a place is dangerous one tends to avoid it, etc.

Part of the content actually conveyed by (1b) is built up by the addressee, having recourse to knowledge he possesses independently from the speaker’s assertions. As a consequence, the addressee will feel that content neither as something the speaker is imposing on him, nor as something which is being proposed to him by the speaker alone; rather, he will feel that content as something he arrived at by himself (Lombardi Vallauri 2009a). For this reason, it is less probable that an addressee will be inclined to question or challenge an implicated content than an asserted one.

### Vagueness

Vague and ambiguous expressions^1^ can potentially refer to more entities or states of affairs:

(2) El Medano Resort: you will enjoy your stay in Tenerife.

Different people planning to book a hotel in Tenerife will imagine different things under the same, generic label “enjoy”. To gourmets, it will evoke exquisite dinners. To playboys, nice girls. To snobs, high society guests. To fans of the sea, a beautiful beach. To others, a nice swimming pool, rooms with many facilities, a tropical garden, perfect service, and so on and so forth. Everyone will be free to think what he wants.

Interestingly, more precise assertions would be more verifiable, leading to more doubts on their truthfulness (“Our dinners are very good”, “You will find many nice girls here”, “We always host VIPs”, etc.). On the contrary, a completely generic, vague assertion such as “you will enjoy staying here” potentially means all of these, but at the same time none of them necessarily. As a result, even if almost all single sub-assertions may be evidently false, the generic assertion cannot be said to be false because it is very difficult to exclude that one of them may be true. A vague assertion is unchallengeable because its meaning potentially all things ends up meaning no single thing necessarily. Nobody can say that some assertion is false, if the assertion is vague enough.

## The similarity between implicits and non-linguistic communication: persuasive effectiveness

As we have just suggested regarding implicatures and vague expressions, the transmission of a content as implicit reduces the probability that the addressee may discuss it. After Gottlob Frege (for ex. Frege 1892: 40) this was noticed by many others (cf. for ex. Givón 1982; Kerbrat-Orecchioni 1986; Rigotti 1988: 118; Lombardi Vallauri 1993, 1995; Sbisà 2007). The issue concerns at least implicatures (Grice 1975; Sperber and Wilson 1986) and presuppositions (Strawson 1964; Garner 1971; Ducrot 1972; Moeschler To appear for a comparison); but also, as we will suggest, some manipulations of Information Structure. This makes implicits one of the cornerstones of linguistic persuasion (cf. Lombardi Vallauri 1995, 2009a; Sbisà 2007; De Saussure 2013; Lombardi Vallauri and Masia 2014).

By definition, it is difficult for linguistic messages to be entirely implicit. But the same is very easy for non-linguistic communication such as images and sounds. Beside obvious reasons of sensorial richness, I would suggest that in persuasive communication (like advertising) images and sounds are considered more effective than textual headlines precisely because they convey their content in a less explicit way. *They do not make statements*. When, on television (as in a famous commercial by Glen Grant), we see a group of young, handsome, rich and happy people drinking some whisky in a wonderful house, to a certain extent we will be influenced by the following idea: “If you drink our whisky, you will be young, handsome, rich and happy, and you will live in a wonderful house”. The same content, *if stated explicitly*, would convince nobody, possibly provoking rather hostile reactions; but in its visual, “implicit”, not-stated version it works very well. The same is true for some musical piece inducing happiness, solemnity etc.: it is by far more effective than any explicit utterance stating the capacity of some merchandise to make you happy, important in the opinion of others, or the like.

The difference between linguistic and non-linguistic messages is that linguistic statements are explicitly conveying information: they reveal that the source has the intention to convince us about certain content. Images and sounds, on the contrary, *do not look as being produced by a human individual*, which gives the addressee the impression that he is free to give them any value he wants, as if there was no commitment in any direction on the part of the source (we will develop this more in detail right away). Crucially, the feeling that the source of the message is trying to modify our status warns us that we should better react critically and check whether the proposed content has to be rejected. On the contrary, the feeling that we are left free to think what we prefer leaves us with the impression that the contents we are exposed to do not need to be challenged.

Given this “explicitness drawback” of linguistic utterances, implicits are the most similar thing to visual and musical communication that language offers, in that they reduce the awareness of the addressee that the source of the message intentionally tries to convince him of some content. When using implicits, language resembles the other components of any multimedia message aimed at persuading its addressees to adopt some (typically, buying or voting) behaviour.

Among linguistic implicits, implicatures and vagueness prototypically represent what we have called *implicitness of content*. We will now suggest that another very important kind of implicit information exists, namely *implicitness of responsibility* (cf. Lombardi Vallauri and Masia 2014).

## Implicitness of responsibility and its “exaptation”

From certain respects more importantly than notional content, in a linguistic message the *assumption of responsibility* for its content on the part of the speaker may remain implicit. We will argue that this is the core feature of *presuppositions* and *topics*.

### Presuppositions as reducers of processing effort

Presupposition consists in presenting some notion as already shared by the addressee(s) (Strawson 1964):

(3) Bob has been unmasked. Sue won’t forgive his cheating.

In (3) the idea that Bob was lying is presented as presupposed by the meaning of the verb *unmask*. The idea that he was not trustful to his wife is also presupposed by the definite description *his cheating*. The speaker presents such contents as if he believes that they are already agreed upon by the addressee, and for this reason he can avoid asserting them. Otherwise he should say something like (4), where each information is first introduced by an act of assertion, and only after that it is referred to via presupposition:

(4) Bob was lying, and they finally unmasked him. He was cheating on Sue, and she will not forgive that.

When a message contains an implicature, the content to be held as true is partly concealed within the folds af the message, i.e. only implicitly conveyed. On the contrary, messages containing presuppositions encode their notional content explicitly, but they conceal the very act of proposing it as true, as if the speaker has no commitment to transferring it. Instead of a world where the speaker wants the addressee to believe something, presuppositions build a world where the addressee is supposed to already know and agree upon that something. In such a situation there is no need to assert that content, but just to resume it for the sake of understanding the rest.

This way to present a content is most effective for the purpose of convincing someone of its truth. As we have already hinted at, if there is something that can cause opposition in humans, it is the recognition of any attempt on the part of someone else to modify their status. Now, that is precisely what defines assertion: asserting means admitting that you consider the addressee unaware, and that you attempt to modify his status into that of a believer. This has been characterized in terms of the participants’ “commitment” (cf. Hamblin 1970 for a general introduction, Morency et al. 2008 for extension to implicit communication, Boulat and Maillat To appear for a new, very detailed definition) or, in the French tradition and terminology, “prise en charge” (cf. Desclés and Guentcheva 2001; Corblin 2003; Beyssade and Marandin 2009). Overt commitment may raise critical reaction, such as “you want me to believe certain content and to commit to it, but exactly because you want that, there is probably some advantage for you and some drawback for me; so I’d better carefully evaluate, and preferably reject that content, not including it into my commitment store”. This is especially true when the addressee has reasons not to trust the speaker, or to suppose that he has some interest or some advantage to be drawn from the addressee, as is typically the case in political propaganda and commercial advertising, as we will see right away.

It is important to understand that, by presupposing a given content, the speaker suggests that *some other situation has caused previous knowledge* in the addressee, so not the speaker, but the other source is responsible for that content. In such a state of affairs, the addressee’s critical reaction towards the speaker has less reason to rise, and may be weaker, or null: there is little need to double check the truth of something we already know about. This effect of what is taken for granted is included by Givón (1982) among the phenomena that he calls “unchallengeability” on the part of the addressee. One is strongly led to treat presupposed content as not subject to possible discussion.^2^

#### The pragmatic path of presuppositions’ “exaptation”

##### Presenting the known as known

Assertion instructs the addressee to treat some piece of information as new to him, to focus his attention on it and work at establishing it as a new piece of knowledge in his mind. Presupposition basically spares this processing effort. When some content is already in the knowledge of the addressee, the speaker should behave accordingly, and present that information as presupposed. This instructs the addressee to just recognize the presence of that content among the things he already knows. In (5), fully assertive constructions are used to present each piece of information as if the addressee was completely unaware of it, resulting in quite unnatural communication:

(5) I exist. I have a father. This father was writing a book. A place called Waterloo exists. A famous battle took place there. My father was writing a book about that battle. There is a month called April. I was married. The day before my wedding fell in that month called April, and my father finished writing that book on that day.

Such assertions instruct the addressee to focus on each mentioned item, and to build a new mental “slot” for the speaker, one for a father he has, one for a book he was writing, one for Waterloo and one for the battle that took place there, etc. Then, he may realize that he already has such slots: in more common words, that he already knows about the speaker, his being born from someone, the existence of Waterloo and its battle, perhaps the speaker’s wedding; let alone about April. This would lead to garden path effects, pragmatic rather than semantic in nature. To avoid such a waste of processing effort, the speaker should better use presupposing expressions, by which he tells the addressee that he is supposed to know already about such contents. This will authorize the addressee not to process such contents in deep detail:

(6) My father has finished his book on the battle of Waterloo in April, the day before my wedding.

If the speaker’s father, the battle of Waterloo or April are encoded as presupposed information (here, by means of definite descriptions), i.e. if the addressee is told that he already knows about the existence of such referents and can identify them, he will avoid unnecessary effort. He will pay *less attention* to that content, because it comes with the suggestion that it is something already known to him, not needing thorough examination any more: full examination of already-known content would be the superfluous repetition of some effort that one has done in the past. A resumptive, “mentally opaque” recollection of the already known (“his father”, “the battle of Waterloo”, “April”) is enough for the purpose of understanding the part of the message which is really new (“he finished the book on that day”).

##### A first “exaptation” step: allowing effort economy on the unknown

In Lombardi Vallauri (2014) it is proposed that, in a discourse perspective, presupposition must be regarded as involving lesser processing tasks as compared to assertion. Reference is made to recent studies trying to inquire this difference through the actual—measurable—efforts displayed in brain activity. The difference between presupposition and assertion (together with that between Topic and Focus) may match that between controlled and automatic processing (Schneider and Shiffrin 1977, 1984) which has been described as follows^3^:

*Automatic* processing is generally a fast, parallel, fairly effortless process that is not limited by short-term memory capacity, is not under direct subject control, and performs well-developed skilled behaviors. […] *Controlled* processing is often slow, generally serial, effortful, capacity-limited, subject-regulated, and is used to deal with novel or inconsistent information. […] all tasks are carried out by complex mixtures of controlled and automatic processes used in combination.

The reason for the existence of double-modality processing can be regarded as adaptive in nature^4^:

Dual processing mechanisms would likely not have evolved unless there were survival advantages to having both modes of processing. […] Automatic and controlled processing are qualitatively different forms of processing that provide complementary benefits. […] A single process alone cannot provide both the fast learning of controlled processing and the high speed parallel robust processing of automatic processing. […] If a task requires the coordination of many sensory/motor inputs, the slow, resource-limited nature of controlled processing will be a serious limitation. Despite taking a long time to acquire, automatic processing has the advantages of being robust under stress, leading to long-term retention of associated skills, and allowing many processes to occur in parallel.

Many other studies point out that controlled processes of our attentional system are strongly affected by limitations, while less limitations arise if, in parallel with controlled processes, some cognitive tasks are carried out automatically. For example, Dux et al. (2006) show that when competing stimuli overlap in central executive processes, only one at a time can be dealt with. Sigman and Dehaene’s (2008) work on so-called “Psychological Refractory Period” evidentiates the inhibition or postponement of the second of two simultaneous tasks. Lien et al. (2006) signal phenomena of Divided-Attention Deficit, i.e. decreasing performances when attention is brought to two simultaneous tasks.

Getting back to language, given the limited amount of resources that can be devoted to each processing task, it is likely that utterances are more ergonomic if they display an informationally hierarchical structure, so as to provide the receiver with instructions on how to distribute his processing efforts more efficiently. Therefore, only some information units in the sentence are realized as prominent (prosodically and syntactically) because they convey the speaker’s informative purpose in the ongoing interaction (Cresti 2000; Lombardi Vallauri 2009b). The receiver is instructed to process such units more carefully. Other units,—typically, the presupposed ones and those in Topic (see below)—will be realized as less prominent because they are less relevant to the communicative task at hand, and the receiver can process them with lesser attention.

So, the existence of means by which, in discourse, some information can be entrusted to automatic instead of controlled processing, is likely to be an obvious advantage in terms of effort economy. Of course, not all information can be processed automatically. Some contents need thorough, controlled processing for their full understanding and in order for them to properly contribute to the comprehension of the whole utterance in relation to its context. Typically, these are referents (1) which the addressee doesn’t know about yet, and (2) whose role in the ongoing discourse is essential. On the contrary, as we have seen, automatic processing is enough for already known contents. As Givón (2002) puts it,

If utterances displayed no differential patterns of prominence and, correspondingly, different informativity degrees, sentence processing would be too demanding for the receiver, as he would be compelled - via extra inferential operations - to calculate the speaker’s intentions in the attribution of prominence statuses to different sentence units. This would tremendously slow down decoding processes and the general unfolding of the conversation. A communication system like this would by no means adapt to the needs of language users, and, most importantly, to the speed at which information transaction takes place in verbal interactions.

Irwin et al. (1982) had already shown that the same nouns tend to be read faster if preceded by the definite article, and more slowly if preceded by the indefinite article, which means that if a certain concept is presented as already known to him, the addressee tends to process it less carefully. More recently, psycholinguistic experiments such as Tiemann et al. (2011), Schwarz and Tiemann (2014), and especially Schwarz (2014, 2015), added strong evidence for the fact that the processing of presupposed contents is faster than that of asserted contents: experimental subjects were faster at answering questions (or fixing eye movements on the right screen images) regarding contents introduced as presupposed, than for contents introduced as asserted. This can be interpreted as probably due to less costly processing of presupposed contents.

Recently, effort economy associated with presuppositions has started being inquired through experimental protocols measuring brain activity (mainly ERP). Not much has been done yet, but the first results confirm (among other things) that the same information is processed with less effort when presupposed than when asserted: cf. in part Burkhardt (2008), and also Bambini et al. (To appear), where we measured ERP reaction of the processing of the same content when asserted and when presupposed in “ecological” stimuli.

To sum up, experimental results seem to confirm that presuppositions instruct the addressee to devote less attention and effort to certain content, because more is not needed for full understanding of the message. Presupposing expressions perform this function, and probably arose in order to fulfil it (Lombardi Vallauri and Masia 2015) for those contents that are already in the knowledge of the addressee. Now, when some feature has developed in an organism for a certain function, humans (as other animals) can devote it to any other function it proves apt to. In evolutionary theory, this is the well-known mechanism which Gould and Vrba (1982) called *exaptation*. As we will see right away, *mutatis mutandis*, pretty much the same seems to have happened for a behavioural trait such as linguistic expressions presupposing their content. Not only the known can be encoded as deserving less attention. As speakers, we are more free than that. We can entrust to less effortful processing on the part of the addressee also new contents, provided they are just marginal information, whose function in the ongoing discourse is not that they must be fully understood, but simply provide a semantic setting for some other information to be fully understood.

If we consider (a): using an expression suggesting to the addressee that he already knows about something he actually knows, and (b): using the same kind of expression to suggest to the addresse that he might treat that piece of information *as if* he already knows about it although he doesn’t; then the path from (a) to (b) is quite a clear one *logically*. It would obviously be much more difficult to make claims about these two stages having appeared in this order *chronologically*, during the evolution of proto-language(s). In any case, real linguistic productions abundantly show that content can be presented as presupposed even if it is not yet in the memory of the addressee. If a speaker has been to Sweden during his honeymoon, he can encode this by a presupposing temporal clause (in italics), like in (7), although he hasn’t told his addressee about that before:

(7) During our honeymoon, *after visiting Sweden,* we spent some time in Denmark.

The same can be said for the presupposition of the change-of-state verb *stop* in (8):

(8) Please, go downstairs and tell May to stop ironing: the oven is also on, and I need to use the vacuum cleaner.

If the addressee is not aware of the fact that May is presently ironing, the speaker might hypothetically be more explicit:

- *May is presently ironing*. Please, go downstairs and tell her to stop, so I can use power for the vacuum cleaner.

Still, (8a) would typically be less natural than (8): asserting information on May’s present activity would result in superfluous effort. The idea that May is ironing can be conveyed as presupposed, exactly as if the addressee already knows about it, together with the request to stop her: in this way, the addressee can devote to that information only the amount of attention which is necessary for understanding the speaker’s request. Utterance (8) is better than (8a) because it costs less processing effort, and draws full attention only where it is really necessary.

##### A second “exaptation” step: smuggling questionable information. Presupposition as a means of distraction

Instructing the addressee not to process some information thoroughly although it is actually unknown to him can have a further function beside allowing him some effort economy: namely, to *prevent him from fully understanding that information*. When certain content is doubtful or even false, the addressee will not accept it if he pays due attention to it. But he may accept it if he remains partially unaware of its most questionable parts, which typically happens if he pays less attention. The fact that some information is doubtful will be evident when it is stated, but may remain unperceived if it is presented so as to be processed more vaguely and less attentively.

A famous psycholinguistic demonstration of this claim is known as the “Moses Illusion Test” (Erickson and Mattson 1981). The aim of the test was to show that the depth of processing of some information may change depending on linguistic “packaging”. A number of subjects were presented with the following question: *How many animals of each kind did Moses take on the Ark?* Almost all subjects responded “two”, without noticing that it was Noah, and not Moses, that took animals on the Ark. The reason for this is that the question *presupposes* Moses having put animals on the Ark, asking only: how many of each kind?

Similarly, Langford and Holmes (1979) measured recognition times of false information when contained in the assertion or in the presupposition of sentences. Consistently with what we are saying, false information was detected faster when it was encoded in the assertion, and slower when it was in the presupposition.

Along the same lines, in Loftus’ (1975) experiments subjects were asked to answer questions on a short film about a car accident. Some of the questions contained false presuppositions, which induced most of the subjects to provide wrong answers, precisely complying with the false presuppositions. In other words, presuppositions proved highly effective in convincing subjects of their contents, even if those contents were in contradiction with what the subjects had witnessed in the film.

**Fig. 1:**
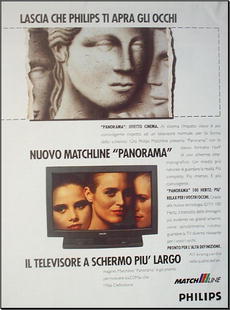
Lascia che Philips ti apra gli occhi

Persuasive communication habits further prove these assumptions, in that they make extensive use of presuppositions in order to “smuggle” doubtful information into the target’s heads. In 1991 Philips diffused an advertisement in Italy, whose headline used to presuppose (by means of the change-of-state verb *aprire* ‘open’*)* that the addressees were living (at least by metaphor) with “closed eyes”.^5^ Its English translation would be:

(9) Let Philips open your eyes (Fig. 1)

The same content would have been received differently if the headline had asserted it: *You are living with closed eyes*! Most of the target would have recognised it as false and offensive. But the ad was designed as it was, because its being presented in the form of a presupposition allowed for the same content to be easily accepted by everyone. This is because presupposition effected its being processed in a vague, less scrupulous way, so that its intrinsic falsity and offensiveness was not noticed. The ad cleverly gives evidence (by asserting it) to the idea of opening one’s eyes. On the contrary the presupposed idea that they were previously “closed” passes into the addressee’s knowledge without undergoing a moment of true focusing, i.e. of purposeful attention, which would probably lead to better awareness and rejection. Lesser attention thus prevents critical challenging of the offensive content, but not its becoming part of the state of the world believed by the addressee after reading the advertisement. In this case, the result is that one feels the strong need to change his “blindness” situation (by buying a new television screen).

**Fig. 2:**
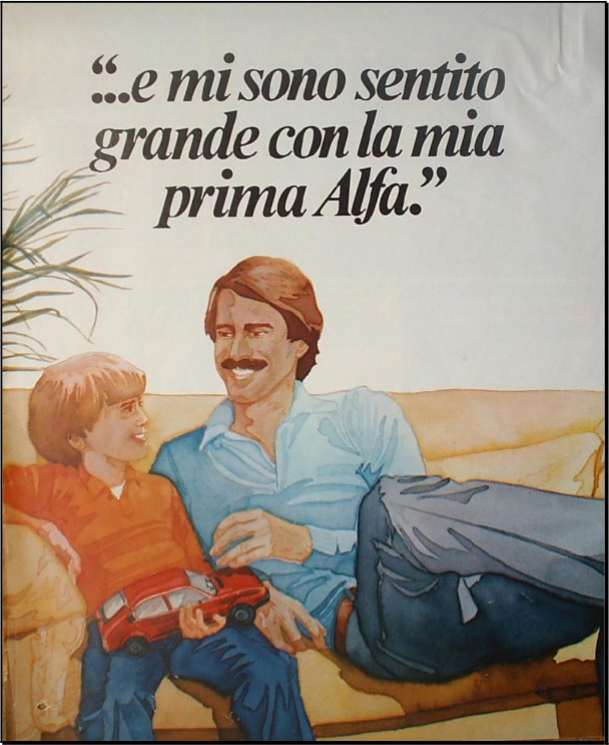
… e mi sono sentito grande con la mia prima Alfa

Presuppositions “silently” drive receivers to conceive of (and reconstruct as existent) portions of reality which are neither in their memory, nor in their general knowledge of the world. Hence, they are effective strategies to “introduce information without calling attention to it” (Loftus 1975: 572). Advertisements massively work like this. In the same period Alfa Romeo showed a happy father telling his son:

(10) “…and I felt grown up with my first Alfa” (Fig. 2)

Introducing the presupposition conveyed by the adjective *primo* ‘first’^6^ is probably the main purpose the copywriters of this advertisement had in mind. Its full content includes:

(11) The happy father has owned several Alfas.

Though apparently the ad just wants to stress the asserted idea of feeling grown up with the car of one’s youth, further contents are more important for its success. Not by chance, the addressee is invited to process such contents less attentively: namely, that the Alfa Romeo owned by the happy father in his youth was followed by more Alfas. This idea, if it is accepted passively and without critical challenging, will silently reshape the set of beliefs which build the mental world of the target, into one where the possession of an Alfa induces people to buy more Alfas. The fact that such a content is highly questionable is bypassed. Now, this content is particularly useful in the view of persuading people to buy Alfa Romeo cars, because it conveys an important inference, again encoding further information implicitly: that who buys an Alfa is usually very satisfied with it.

#### The threefold function of presuppositions

Summarizing, we have seen that *instructing the addressee to pay less attention* to certain content is the general processing correlate of presupposition in discourse. But its purposes can vary according to the different statuses the presupposed contents have in the mind of the addressees when they are uttered:

- **Purpose 1**, when the content which is presented as presupposed is actually shared and already known to the addressee:
  **to save the addressee the superfluous effort which would result from processing that content*ex novo***;
  **Purpose 2**, when the content which is presented as presupposed is not actually shared or already known to the addressee, but it is not questionable, and it is *bona fide* true:
  **to save the addressee the superfluous effort of processing content that can receive minor attention without any damage to the comprehension of the message**;
  **Purpose 3**, when the content which is presented as presupposed is not actually shared or already known to the addressee and, in addition, it is questionable or even false:
  **to prevent the addressee from becoming completely aware of the details of that content, lest he may challenge and reject it.**

The path from its basic function, represented by Purpose 1 (processing economy on known items), to Purpose 2 (processing economy on unknown marginal items), is thus only the first step of what we have called (by metaphor) the “exaptation” of presuppositions (and, as we will see right away, other linguistic implicits). The second step is represented by Purpose 3, where economy of effort is no longer the ultimate reason why implicit constructions are used, rather it is just a device to obtain partial distraction, and persuasion.

### Topic status as a weak means of distraction

The information status of being a Topic shares with that of presupposition the fact that it lacks assertive force (Cresti 2000; Lombardi Vallauri 2009b). Presupposing allows to bypass the first introduction of certain content, by assuming that it has been already introduced to the addressee by some previous circumstances, including circumstances extraneous to the ongoing discourse. By presupposing, the speaker either *recognizes* that some information is present in the Long-Term Memory of the addressee, or he *pretends* that this is the case. By assigning the status of Topic to certain content, the speaker suggests that he considers it *Given* information (Chafe 1987, 1992), i.e. presently *active* in the hearer’s Short-Term Memory, because it has been just introduced by the preceding discourse or by its extralinguistic context. On the contrary, being in Focus presents certain content as *New* information, i.e. presently *inactive* in the Short-Term Memory of the addresee(s). This condition provides the reason why the Focus coincides with the illocutionary aim of the utterance (Cresti 2000): utterances are produced to convey information the addressee does not yet possess. In (12) and (13), which are one the reverse of the other, only the Focus is asserted, while the Topic just provides non-asserted semantic background by resuming information which has just been activated by the preceding turn (suggested in brackets). This, as can be seen, overcomes the syntactic structure of the utterance, so that in (13) the content asserted by the utterance is not that of the (topical) main clause, but that of the subsequent, focal subordinate clause:

As a consequence, by definition (Cresti 1992, 2000; Lombardi Vallauri 2001, 2009b) the content of a Topic is not asserted, but just resumed in order to provide the necessary semantic frame for the understanding of the Focus, which carries the illocutionary force of the utterance. This does not completely exclude the responsibility of the speaker for the introduction of the considered content, because he may have encoded it by assertion just a moment before; but at least it reduces his responsibility within that utterance. And previous utterances are already in part forgotten, so that responsibilities stemming from them are rather vague. But, more important and parallel to what happens with presuppositions, some content can be topicalized also in case the first introduction by the speaker has never taken place. Also in this case, and similarly to what happens with presuppositions, this leads to more probable acceptation of questionable, doubtful contents, because Topic status suggests the addressee to pay less attention to the details of that piece of information, which is presented as having been already introduced in discourse and being already part of his knowledge.

This was already pointed out in Bredart and Modolo’s (1988) manipulation of the Moses Illusion Test. They changed the syntactic structure of the original sentence as to have *Moses* once in Focus and once in Topic position (cf. *It was* [MOSES]_F_*who took two animals of each kind on the Ark*, vs. *It was* [TWO ANIMALS]_F_*of each kind that Moses took on the Ark*). Not surprisingly, the experimental subjects noticed the distortion when *Moses* was in the sentence Focus, while they tended to miss it when it was conveyed as Topic, that is in the complement clause of the cleft construction.

This hypothesis is further strengthened by studies like Birch and Rayner (1997), showing that reading times for topical information (measured through eye movements) are faster than for focal information. This is commonly interpreted as the Topic receiving less attentive processing. In change-detection tests (Sturt et al. 2004), sets of sentences were manipulated, so as to have lexical changes once in focal, once in topical position. The changes were more easily detected when they involved the sentence Focus. Once again, the most probable reason why Focus facilitates the recognition of lexical substitutions or anomalies of any kind, is that it instructs to more attentive processing.

However, Ward and Sturt (2007) showed that lexical substitutions are frequently recognised also when presented in what they defined *implicit* ways of presentation, because—in their explanation—the cognitive system can register certain content “without this information reaching the level of conscious awareness”.

As for neurolinguistic studies, much remains to be done. According to Burkhardt and Roehm (2007) and Benatar and Clifton (2014), New contents require the *updating of the register*, i.e. that *new slots are created* for them in memory, while Given contents essentially require the *linking to the already existing register*, i.e. the *recognition* of referents that already exist in memory. This raises the hypothesis (Lombardi Vallauri and Masia 2015) that IS categories, and in particular Topic and Focus, have developed in all languages to ease the processing of upcoming information by signaling which of these two functions each chunk of information must undergo. Topical packaging has the main function of telling the addressee to *recognize* certain content just by looking for instances of the same information among the concepts that are presently active in his memory. Focal packaging, on the contrary, instructs to consider some information as a fresh contribution of that utterance, needing for *anew position to be created* in memory. Such different tasks are likely to require processing efforts that are different in nature, and probably also in intensity.

In fact, Wang et al. (2011) measured more prominent N400 effects for focal words as compared to topical words, suggesting that more attentional resources are allocated for Focus units, and Topics are processed in a more “shallow” manner. In La Rocca et al. (2016) we have shown that focused information—no matter if contextually Given or New—determines higher amplitudes in the *θ* rhythm as compared to topical presentation of the same notional content, which may confirm that Focus triggers more processing effort than Topic.

This property of Topic-Focus Structure is (by instinct or specific knowledge, we don’t know) typically exploited by professionals of persuasive communication. In utterances like (14) and (15), from political speeches by Matteo Renzi and Paola Taverna, the part which is presented as a (preposed or postposed) Topic encodes information which the speaker prefers not to present as introduced by himself, but rather by the circumstances: something whose activation in the ongoing discourse is not due to his responsibility, and which he is somewhat obliged to resume because it is already at issue:

(14) Dall’altro lato, un’idea di Europa che in questi anni non ha funzionato, ha fallito.
  *On the other side,an idea of Europe which hasn’t worked in these years*, *has failed*.
(15) Insomma un delinquente abituale, recidivo e dedito al crimine, anche organizzato, visti i suoi sodali.
  *In sum, a habitual offender, recidivist and devoted to crime, even organized,seen his friends*.

In (14), the “fact” that a certain idea of Europe hasn’t worked is in Topic, i.e. presented as Given information. This produces the impression that this is not just the speaker’s fabrication, rather a state of affairs proposed by the actual circumstances and consequently already active in the hearers’ consciousness. The same holds for the idea of “who his friends are” in (15): their connection to organized crime is presented as already present in the hearers’ Short-Term Memory, i.e. put forward by the general situation, not by some malicious insinuation on the speaker’s part. This is likely to trigger less attentive processing, and more probable acceptation.

## High diffusion of a message reduces its challengeability

The distracting effect of implicits becomes stronger in communication directed to vast audiences. In face-to-face interactions the addressee knows that the choice to challenge any content conveyed by the speaker entirely rests upon him. For example, if in his opinion the mentioned idea of Europe hasn’t failed, the hearer of (14) may challenge the presupposed content, expose the presupposition and dissociate himself from any assumption on his supposed sharing the speaker’s beliefs (von Fintel 2004; Pearson 2010): *But wait a minute: that idea of Europe has given great results!* Moreover, and very importantly, the hearer knows that if the presupposition is false, no one else than himself can expose it.

On the contrary, in public communication (such as advertising or propaganda), a target of very many people is reached. This means that, for instance, a presupposed content is presented as already shared and agreed upon by very many people. And obviously, nobody stands up to challenge it. Nobody says to Philips: *Come on, I am not living with closed eyes!* In fact, there is no actual possibility for someone to publicly protest against a printed or a broadcasted advertisement, saying that he does not share the presupposition. Still, a sort of confirming silence on the part of a vast audience, possibly up to millions of people, is not without effect. As we have seen, the effectiveness of presuppositions depends on the fact that there is little need to double check the truth of something one already knows about: obviously, there is even less reason to double check something *everybody already knows about*. This results in a *compelling silence*, because each single person who is reached by the advertisement feels “too little” to critically challenge a content which is apparently shared and agreed upon by so many people.

This proves extremely useful for persuasive communication. We would like to suggest that the “authority effect” of mass media, which is well known in general, becomes even more effective when it comes to the implicit side of communication. If the source of an utterance is an important public organization such as a newspaper or a television broadcasting company, “undermining the speaker’s authority to produce that utterance” (Sbisà 2007) becomes really difficult.

## Some evidence from Italian commercial advertising and political propaganda

Advertising and political propaganda have among their pillars the exploitment of implicits, along the lines we have introduced here. We will show some examples of Italian commercials and announcements from the political campaign 2006. Not rarely, a single advertisement can exploit more than one kind of implicit.^7^ We will group them according to the main strategies they employ.

### Exploitation of implicatures and vagueness

**Fig. 3:**
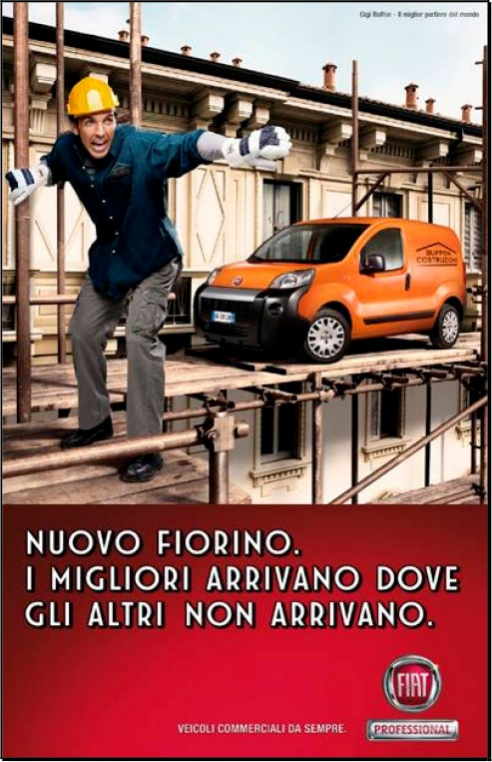
“Nuovo Fiorino. I migliori arrivano dove gli altri non arrivano” (New Fiorino. The best arrive where the others don’t)

In Fig. 3, Fiat exploits the Gricean Maxim of Relevance in order to have the addressee establish—by implicature—a relation of reciprocal relevance between the name of the product (*Nuovo Fiorino*) and an assertion which is left without any explicit syntactic or semantic link to it.

This proceeding is made more efficient by the fact that the content to be implicated—and believed—is very *vague*. If it was that Nuovo Fiorino has the most powerful engine, or the safest brakes, or the most beautiful design, its questionability would be much more evident. But what does “being the best” mean? It is so generic, it can be taken in so many senses, that it is really difficult to think it a *wrong statement*. This is the power of vagueness.

**Fig. 4:**
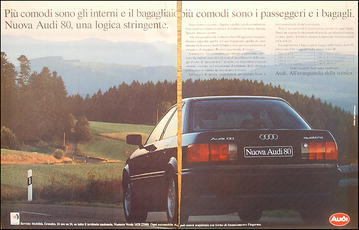
“Più comodi sono gli interni e il bagagliaio, più comodi sono i passeggeri e i bagagli. Nuova Audi 80, una logica stringente” (The more comfortable the interiors and the luggage van, the more comfortable the passengers and the luggage. New Audi 80, compelling logic)

An implicature is at work also with the rather complex and captivating reasoning in Fig. 4, whose relation with Audi 80 is not stated, but left for the target to implicate as a consequence of the bare citation of the car’s name: If the message has to respect the Cooperation Principle,^8^ there must be a relation between its statements and Audi 80. The double meaning of *comodo* ‘comfortable’ if attributed to container and content is noteworthy, and ultimately distracts the addressee from noticing that the whole discourse is so general and necessary in nature that it could apply to *any* car, not only to Audi 80.

In both cases, the assertion is a truism and not relevant in itself: the aim of the message cannot be to convey its content. This is an extremely frequent proceeding in advertising: the relevant content is obtained via *asyndeton*, by simply juxtaposing two other contents. These can be expressed both by linguistic utterances, but also by different parts of the message: typically, images. This is what happens for instance in the Glen Grant spot we have commented on above, where the relation between a brand of whisky and the happiness/beauty/wealth etc. of the people who drink it is not stated, but expressed by simply putting the name of the product close to the images: the target does the dirty job, i.e. decides that there is a relation between the two. But his “decision” is strongly conditioned by the Cooperation Principle, because the whole message would end up being infelicitous if that relation doesn’t hold. The implicatures arising from the utterances in Figs. 3 and 4 are: “Nuovo Fiorino is the best” and “Audi 80 has the most comfortable spaces for your comfort”. The people reading the ads build them by themselves, under the influence of the Cooperation Principle: as a consequence, they are led to accept them more easily than they would if such a statement was formulated explicitly by Fiat or Audi.

**Fig. 5:**
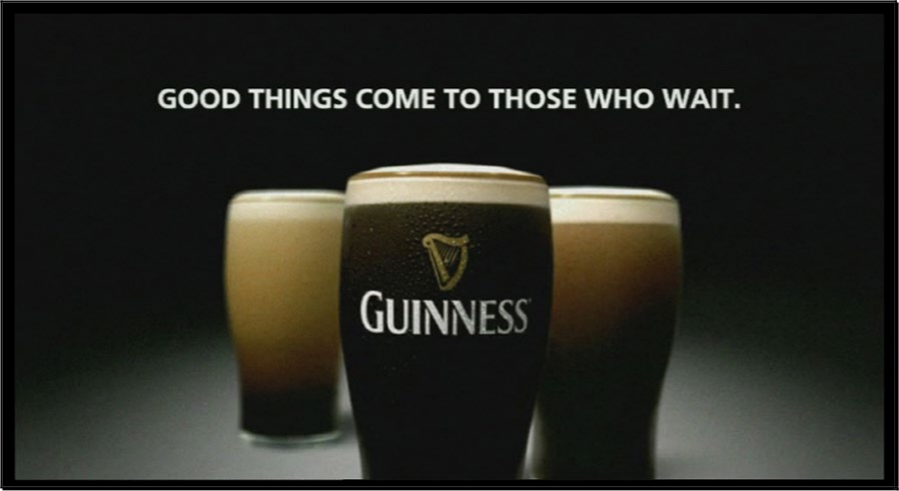
Good things come to those who wait

A similar exploitation of the implicature arising from asyndetical juxtaposing (including images) is in Fig. 5.

Once again, the rather trivial and vague assertion of the headline is not explicitly referred to the depicted beer by the headline, but by the target himself, who is less tempted to doubt about the idea that Guinness is a “good thing”, than if the same idea was asserted explicitly by the producer. Not by chance, the ad was diffused in English in Italy, because the essential of it was not the perfect understanding of the wording, but just that the target could recognize the meaning of the English words *good things*, and relate them to the image of the beer.

**Fig. 6:**
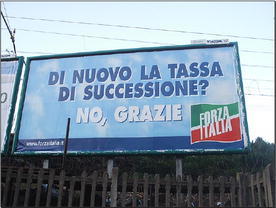
Inheritance tax again? No, Thanks

**Fig. 7:**
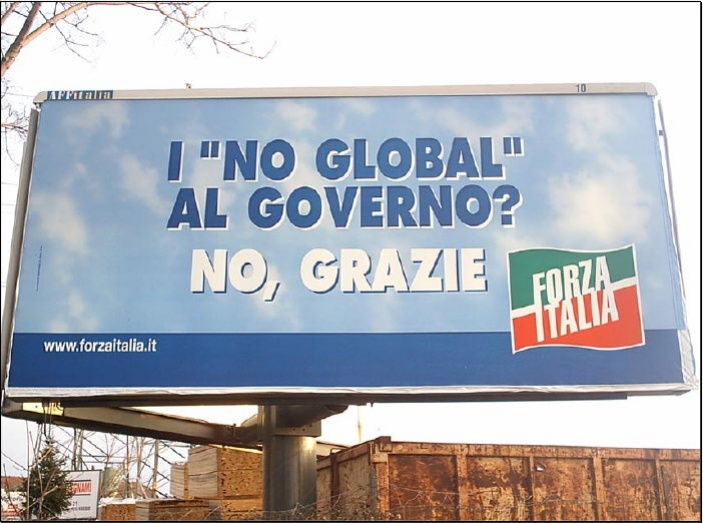
The “no globals” in the government? No, Thanks

**Fig. 8:**
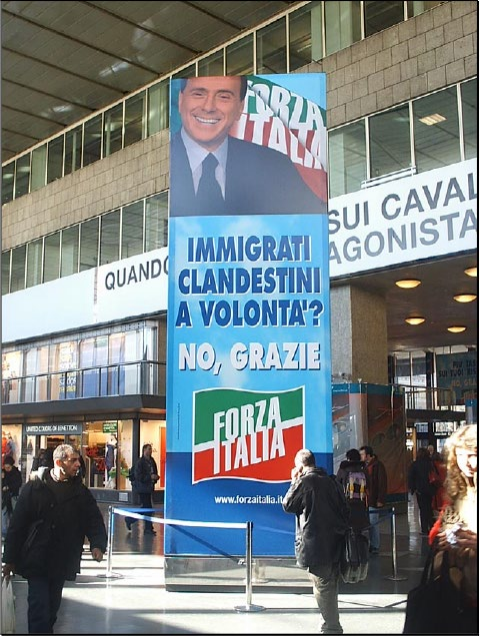
Illegal immigrants at will? No, Thanks

**Fig. 9:**
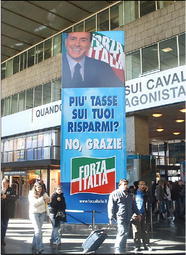
More taxes on your savings? No, Thanks

**Fig. 10:**
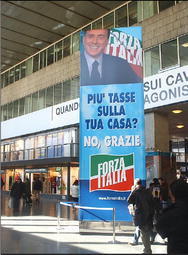
More taxes on your house? No, Thanks

**Fig. 11:**
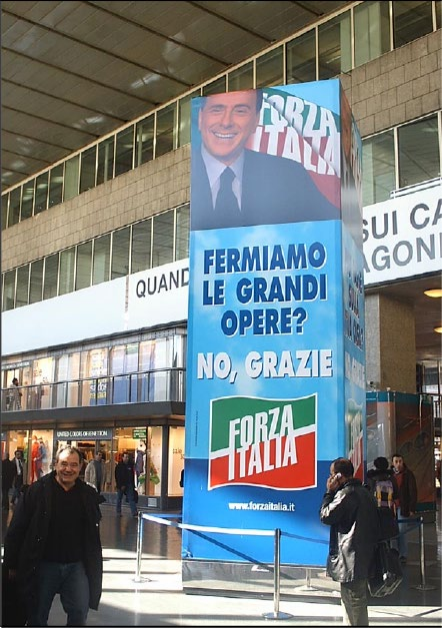
Halting major works? No, Thanks

 Political announcements in the 2006 national campaign in Italy were completely built on the persuasive effects of implicatures. These were the messages diffused by the Right coalition (Figs. 6, 7, 8, 9, 10, 11).

As can be seen, all announcements systematically exploit the same structure. They mention a threatening hypothesis, and explicitly reject it. Still, the most important content of each message is not that the Right gives up that hypothesis: much more important and effective is the implicature that the Left, if given power on the nation, would put those hypotheses into effect. This could by no means be asserted directly, because the Left had not included those hypotheses (at least not in such a bold form) in its program; and in any case directly formulating such accusations would have put the Right in a bad light. But since the mentioned hypotheses were typical stereotypes of a leftist attitude, they could be used as very effective implicature triggers. And as implicatures they worked pretty well, because the readers would conceive autonomously the idea that the Left had the intention to do harmful things, and they would not attribute the responsibility of that idea to a malicious attitude on the part of the Right.

Once again, it can be observed that some messages are made even more effective by their vagueness. What could “at will” mean regarding the entrance of illegal immigrants? At whose will? What should “your house” mean? Some real estate investment, which leftist policies tend to tax energically, or the so-called *prima casa* (first home), which leftists usually protect from taxing? What were the “major works”? High speed trains? The bridge between continental Italy and Sicily in Messina? Others? The Left may have different opinions on each of these, but vagueness allowed for the readers of those messages to implicate that they were all to be stopped in case of a leftist victory. Implicatures and vagueness made it possible to convey the unassertable, and possibly to convince millions of people about it.

The Left coalition used exactly the same (linguistic) strategy.

**Fig. 12:**
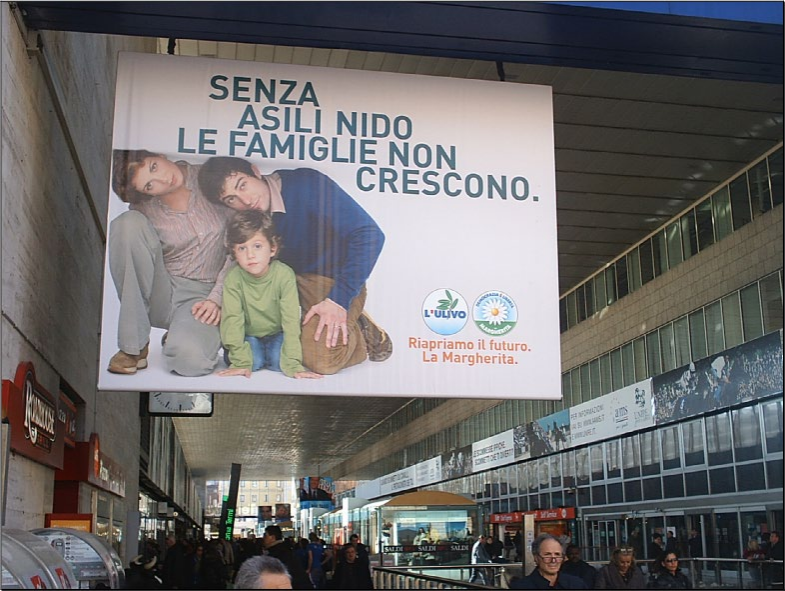
Without nursery schools, families can’t grow

**Fig. 13:**
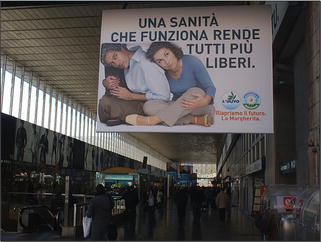
A public health service that works means more freedom

**Fig. 14:**
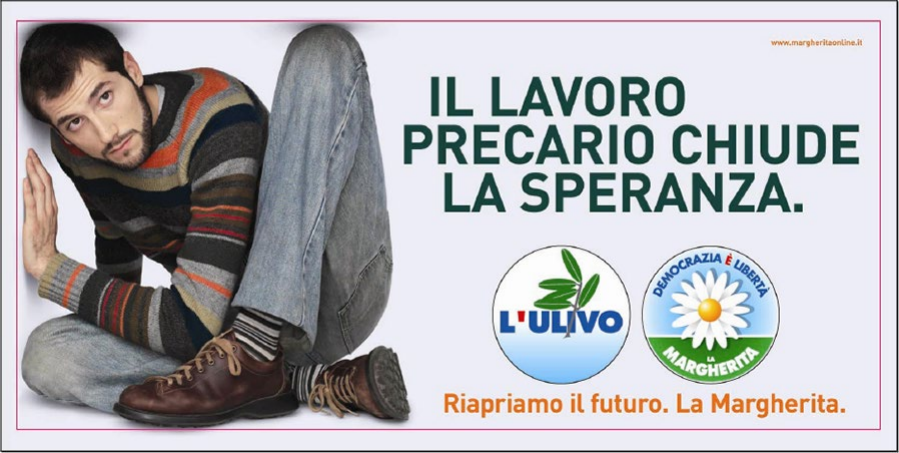
Temporary work clamps down your hopes

As in the rightist announcements, here also we have the exploitment of Gricean implicatures, resting on stereotypes of what was likely to happen if the opponents would win the elections. The truisms in Figs. 12, 13 and 14 are too obvious to build a cooperative message on their own, so—capitalizing on what is more likely—readers would draw the implicatures that the Right was about to cut on nursery schools and public health services, without explicit assertions on the part of the Left, which would have been unpleasant and questionable (the Right having not announced such initiatives), and consequently counter-productive, if directly stated.

**Fig. 15:**
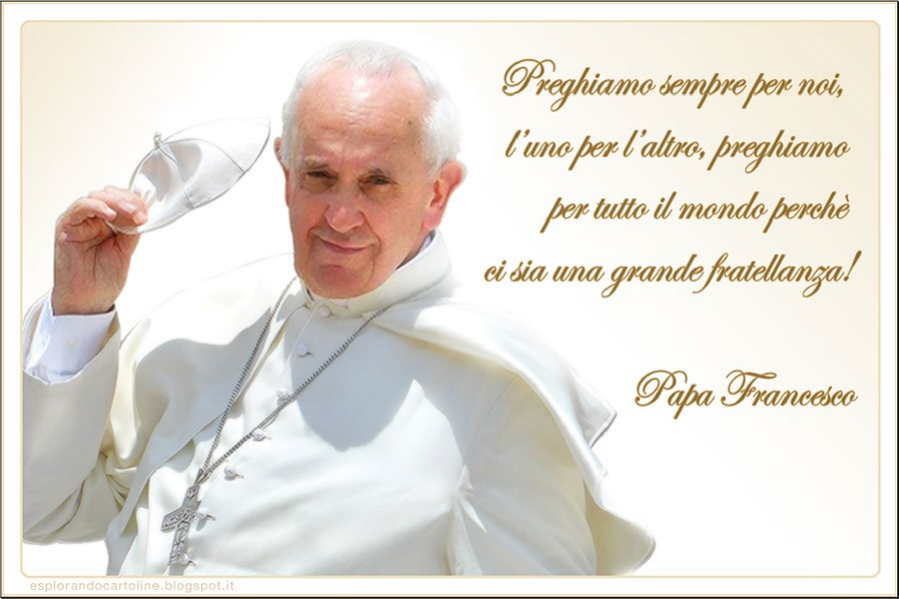
“preghiamo per tutto il mondo perché ci sia una grande fratellanza!” (Let us pray for the world, so that there may be wide fraternity!)

The Catholic advertisement in Fig. 15 invites to pray for fraternity across the world, and meanwhile it exploits the Gricean Maxim of Relevance to implicate a crucial notion (and a quite difficult one to believe) of that religion, namely that prayers are effective.

**Fig. 16:**
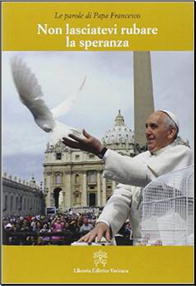
“Non lasciatevi rubare la speranza” (Don’t let them steal your hope)

**Fig. 17:**
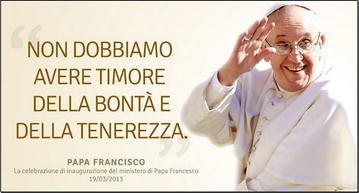
“Non dobbiamo avere timore della bontà e della tenerezza” (We must not be afraid of goodness and tenderness)

Something similar is effected by the ads in Figs. 16 and 17.

The suggestions contained in the Pope’s words are quite easy to agree with, but meanwhile they take for granted as their felicity conditions, and/or convey by Gricean implicature, rather questionable opinions such as:

- we fear goodness and tenderness;
- there are people (typically not Catholic) who try to steal your hope;
- it is easier to hope and to experience tenderness if one follows the Catholic religion.

**Fig. 18:**
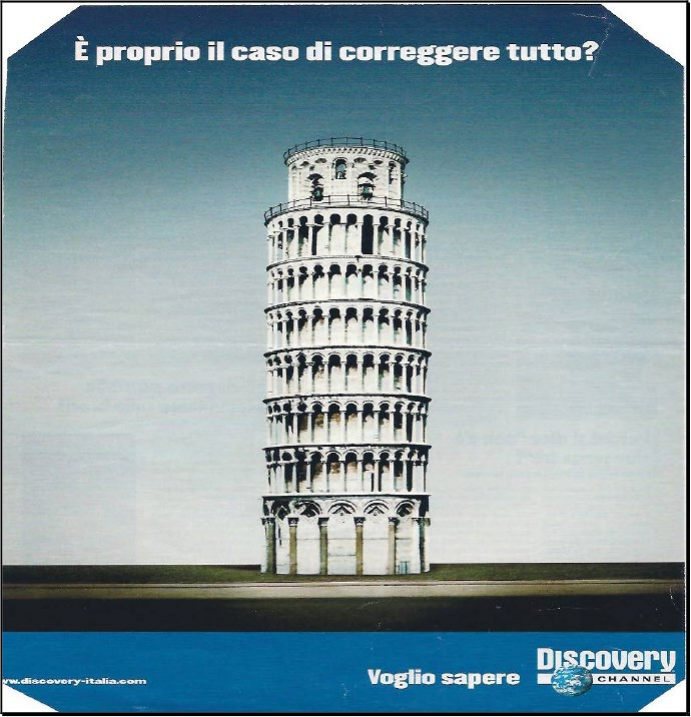
“È proprio il caso di correggere tutto? Discovery Channel” (Is it really necessary to correct everything?)

**Fig. 19:**
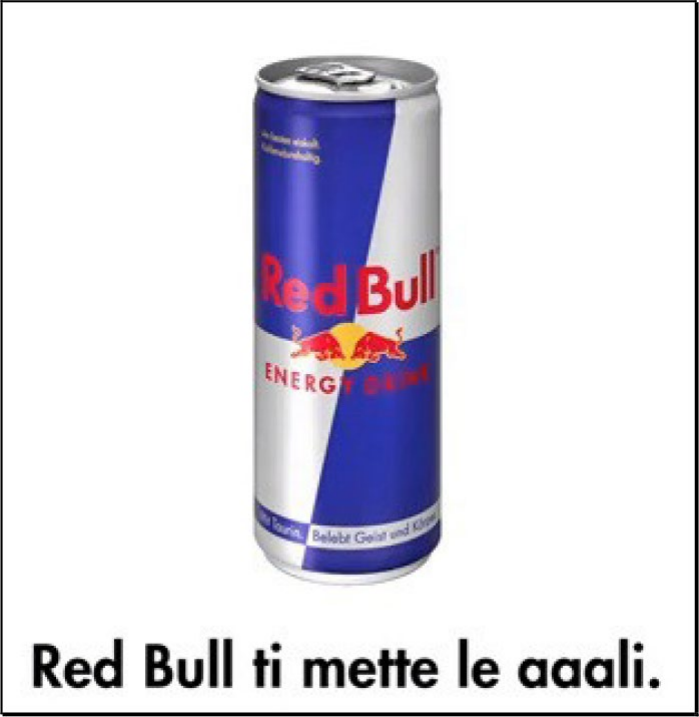
“Red Bull ti mette le aaali” (Red Bull gives you wiiings)

The headline in Fig. 18 exploits the Maxims of Quantity and Relation to silently implicate that Discovery Channel does not show reality different from how it is, and—even more important but much harder to assert, that other channels do so. Even advertisements that may look quite direct are hardly exempt from some tacit assumption. For example, in Fig. 19, Red Bull implies *that you drink it* (otherwise it could not make any effect on you); and its assertion (“gives you wiiings”) is so vague and metaphorical that it is virtually impossible to find it false.

**Fig. 20:**
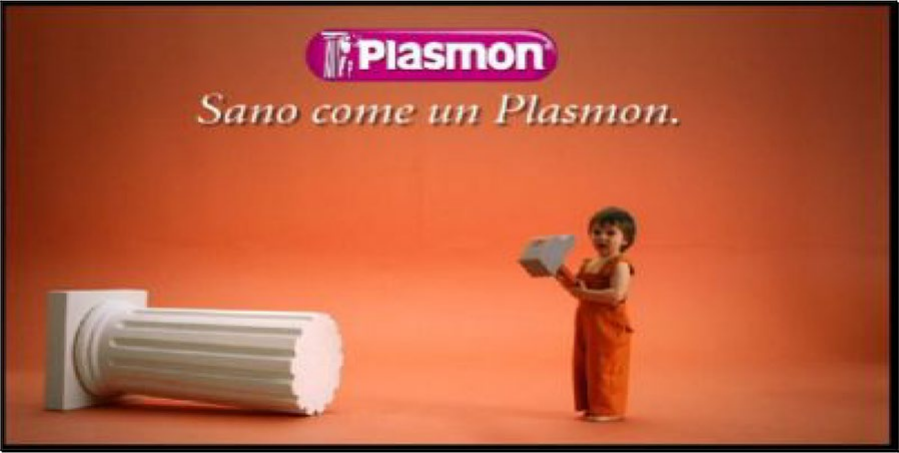
“Sano come un Plasmon” (As healthy as a Plasmon)

**Fig. 21:**
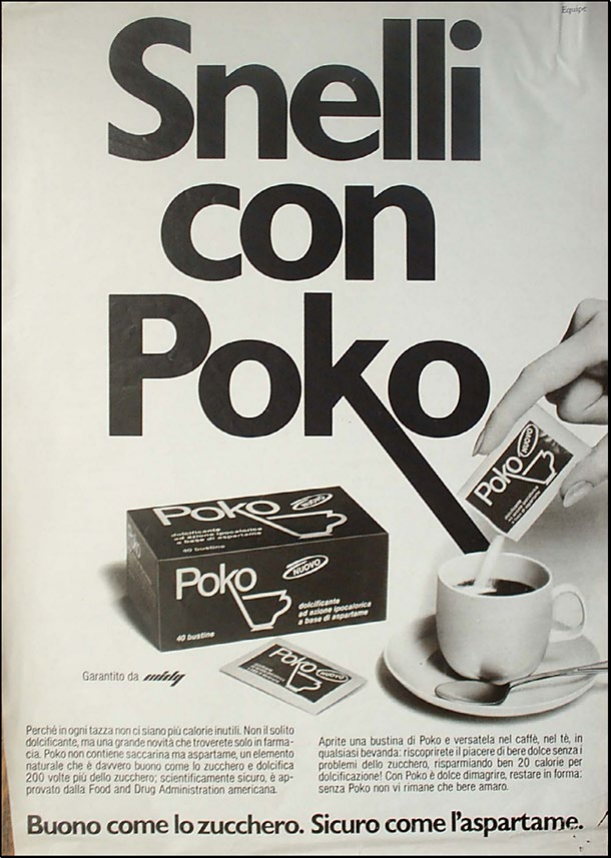
“Buono come lo zucchero. Sicuro come l’aspartame” (As good as sugar. As safe as aspartame)

In Fig. 20, the comparative construction (cf. Lombardi Vallauri 2000) presupposes that Plasmon is healthy, and suggests the implicature that it is among the best possible examples of healthy food, which would be hard to assert explicitly. The same is done with aspartame in Fig. 21, which is remarkable because the ad was diffused (back in the eighties) when aspartame suffered from the suspect of causing cancer. Obviously, the direct statement that aspartame did not cause cancer would have been a marketing *harakiri*; and the unrequested claim that it was healthy wouldn’t have done much better. But its being *implicitly* proposed as the prototype of healthy stuff worked pretty well. It is also interesting to notice that the effect was reinforced by means of sophisticated rhetoric: putting together two hemistichs, formally identical, suggests that they are also identical in truth value: since, in the first, sugar is a widely recognised, typical example of a well-tasting food, this exerts a sort of “similarity-effect” on the second, helping aspartame to be felt as a very typical and recognised example of healthy food.

**Fig. 22:**
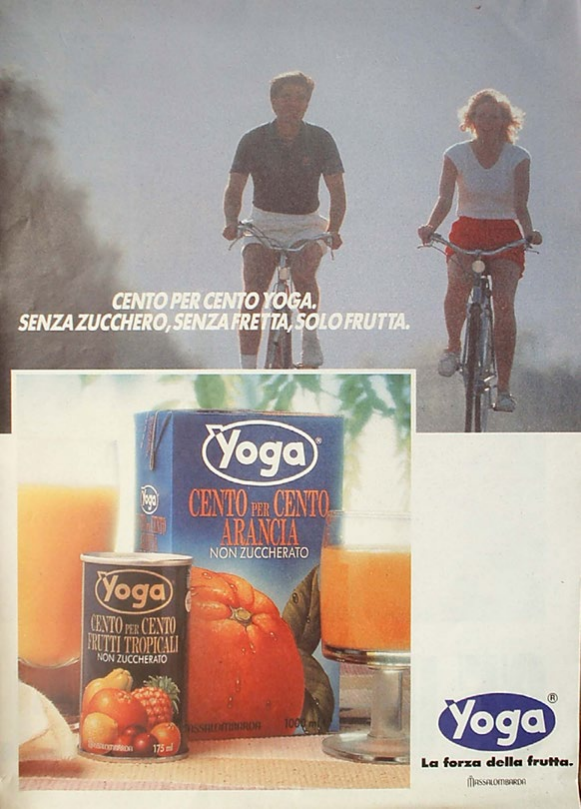
“Cento per cento Yoga. Senza zucchero, senza fretta, solo frutta.” (Hundred per cent Yoga. No sugar, No hurry, Just fruit)

**Fig. 23:**
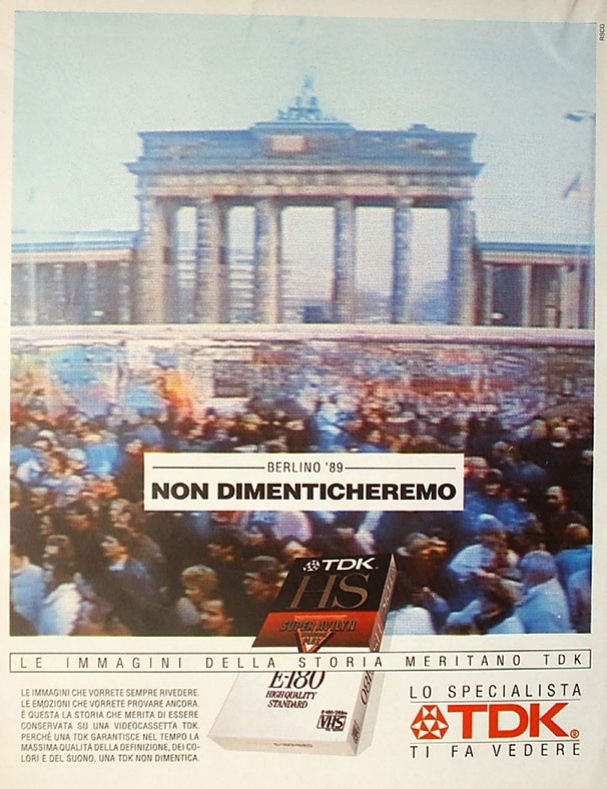
“Le immagini della storia meritano TDK” (The images of history deserve TDK)

In the same vein, “100 % Yoga” in Fig. 22 conveys by implicature something that would be hardly assertable, namely that Yoga is the utmost desirable content of a fruit juice pack (the usual claim would be: “100 % fruit”. In Fig. 23, “The images of history deserve TDK” implicates the idea that TDK are among cassettes the equivalent of the end of the Cold War in history. Asserting the same idea would sound boasting and exaggerate, but if it is conveyed by implicature through a “reversed” attribution of suitability like that in Fig. 23, it can work and convince.

### Exploitation of presuppositions

**Fig. 24:**
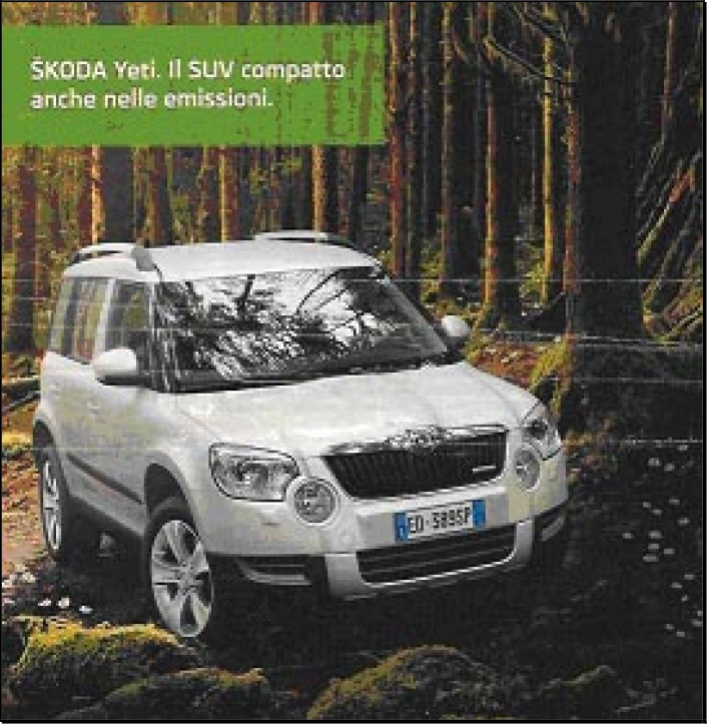
“Škoda Yeti. Il SUV compatto anche nelle emissioni.” (The SUV which is compact also in emissions)

**Fig. 25:**
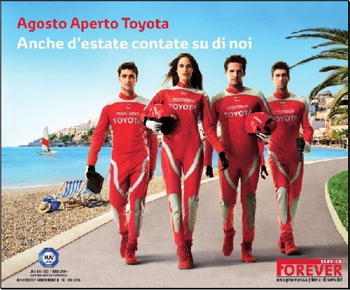
“Agosto Aperto Toyota Anche d’estate contate su di noi” (Open August Toyota. Also in summer, count on us)

So far we have mainly seen implicatures and vagueness at work. But presuppositions are even more important in advertising. Lexical items conveying some presupposition are of constant use. In Figs. 24 and 25 the adverb *anche* presupposes ideas that would be less convincing if asserted, namely that Škoda Yeti is compact in dimensions, price etc., and that you can count on Toyota *all the time*.

**Fig. 26:**
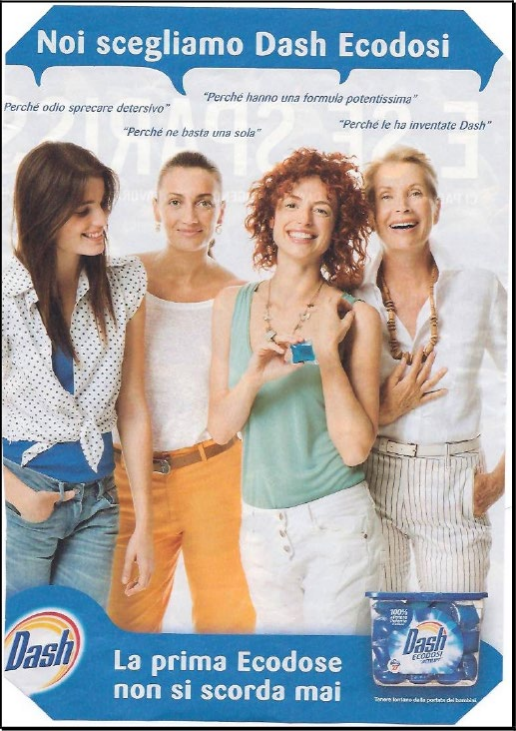
“La prima ecodose non si scorda mai” (One never forgets the first ecodose)

**Fig. 27:**
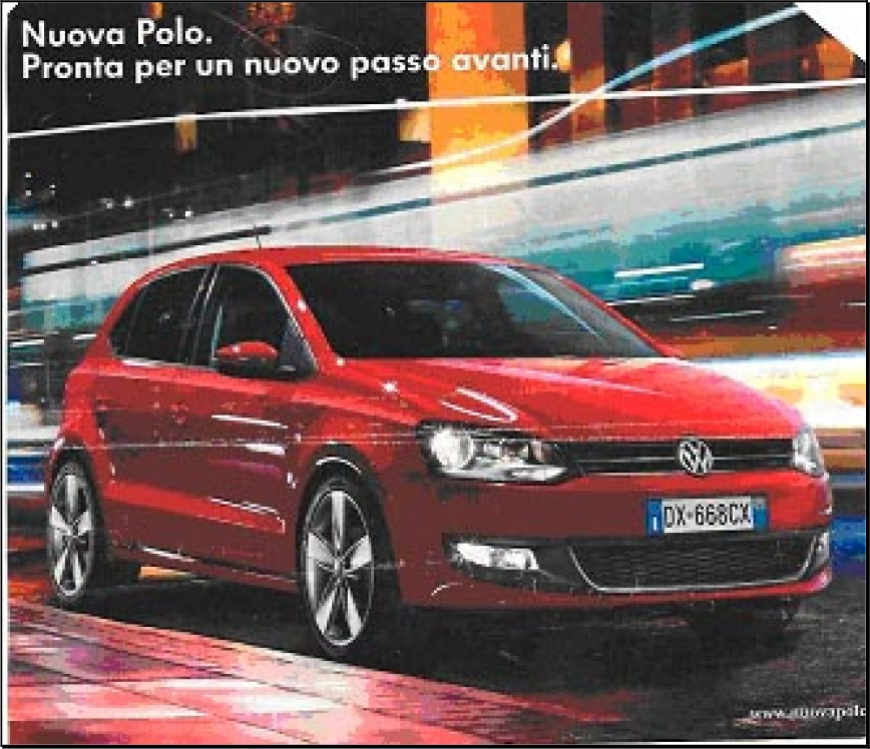
“Nuova Polo. Pronta per un nuovo passo avanti.” (New Polo. Ready for a new step forward)

In Fig. 26, while it is quite frivolously asserted that The first Dash Ecodose cannot be forgotten, the adjective *prima* ‘first’ (cf. also Fig. 2) presupposes that after trying that product one goes on using it. Due to the adjective *nuovo* ‘new’, which presupposes the existence of previous instances of what it describes, the Volkswagen headline in Fig. 27 proposes explicitly that Polo is ready for a new step ahead (whatever this very vague and very positively connoted expression may mean), and presupposes that this is just the last of a series of similar steps made in the past.

**Fig. 28:**
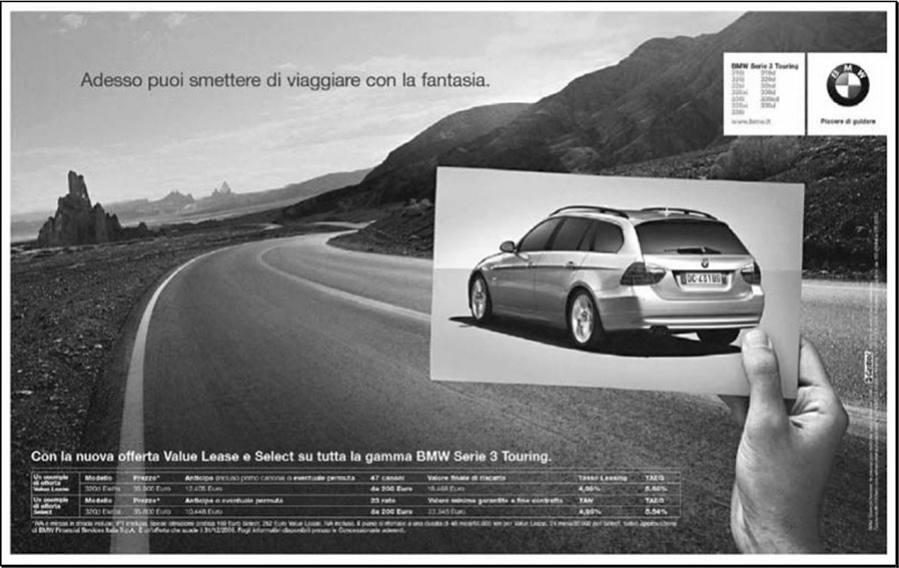
“Adesso puoi smettere di viaggiare con la fantasia.” (Now you can stop traveling with your fantasy)

The egressive verb *smettere* in Fig. 28 presupposes that you were traveling only with your fantasy, which tacitly means that traveling with your present car is not traveling for good. The use of the verb *puoi* ‘you can’ lets you implicate that you want to get out of that condition. The mere, asyndetical presence of a BMW in the picture raises the implicature that BMW will save you from all that. All of this would have been quite hard to convey to the addressees through assertions, but can be smuggled safely through implicit constructions.

**Fig. 29:**
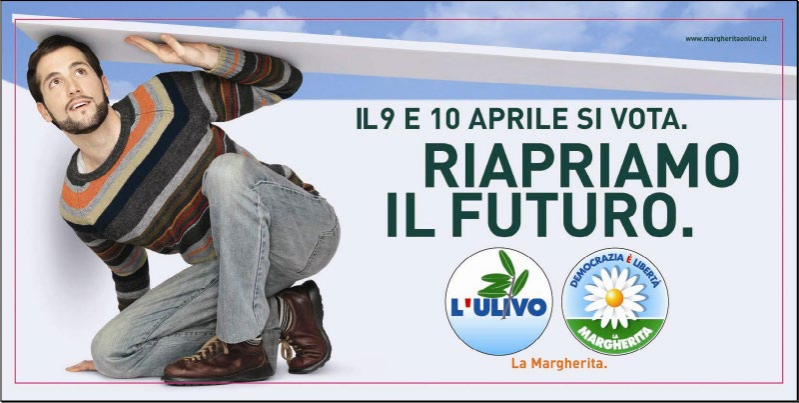
Let’s re-open the future

**Fig. 30:**
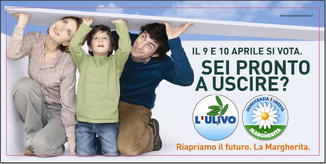
Are you ready to come out?

Also the following announcements (from the Italian leftist political campaign 2006) exploited change-of-state verbs (Figs. 29, 30).

At the time, Berlusconi was ruling. Asserting something like “the future is closed” or “under this government we are imprisoned” would have been excessive and poorly convincing even if addressed to people of strong leftist sympathies. Since the announcements were aimed mainly at convincing those who still had to decide, that content needed to be conveyed in a less explicit way. Which is what the copywriters of the Left actually did.

**Fig. 31:**
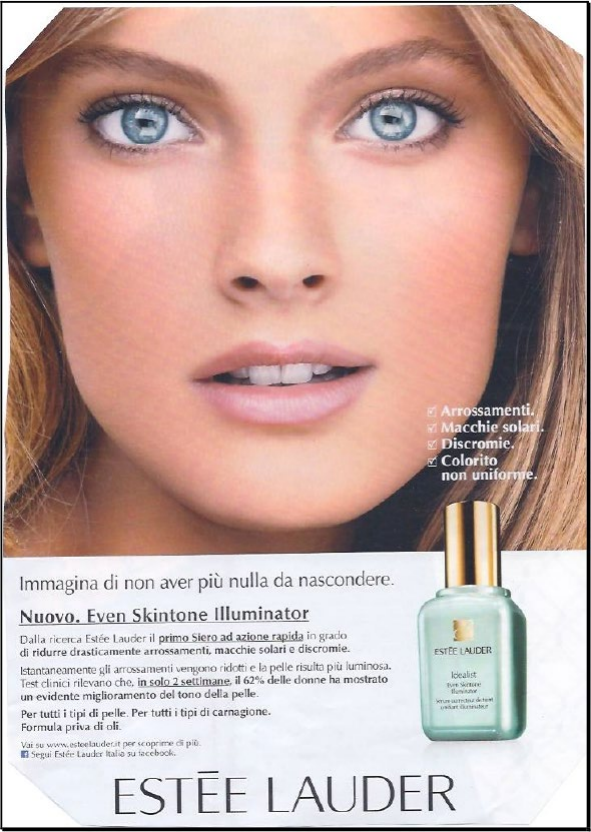
“Immagina di non avere più nulla da nascondere. Nuovo. Even Skintone Illuminator Estée Lauder” (Imagine you have no more things to hide. New. Even Skintone Illuminator Estée Lauder)

In Fig. 31, the following assumptions count among the felicity conditions for Estée Lauder’s headline “Imagine you have no more things to hide”:

- So far, your skin had imperfections you needed to conceal;
- You used to mask them in a way or another;
- *Even Skintone Illuminator* allows you to stop masking them, because it eliminates them.

All this, though not asserted, belongs to the message, by means of presupposition, “freely” but obediently recognized by the target.

**Fig. 32:**
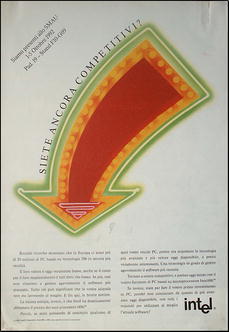
“siete ancora competitivi?” (Are you still competitive?)

In Fig. 32, the adverb *ancora* ‘still’ presupposes that the target’s firm used to be competitive. This makes the perspective of not being so significantly more painful than if one has never been: loosing something we used to own is much worse a sensation, than having simply never possessed it. Now, the quite apodictic assertion that “you used to be competitive” would be likely to encounter critical reaction and awareness about its being inappropriate on the part the addressees, with consequent least impact of the advertisement; but its presupposition can induce fear of losing the status of being competitive even in people who in the past never thought about being so. The solution to such a danger is proposed, not by assertion but by implicature, triggered by the mere presence of its name on the same page: Intel.

**Fig. 33:**
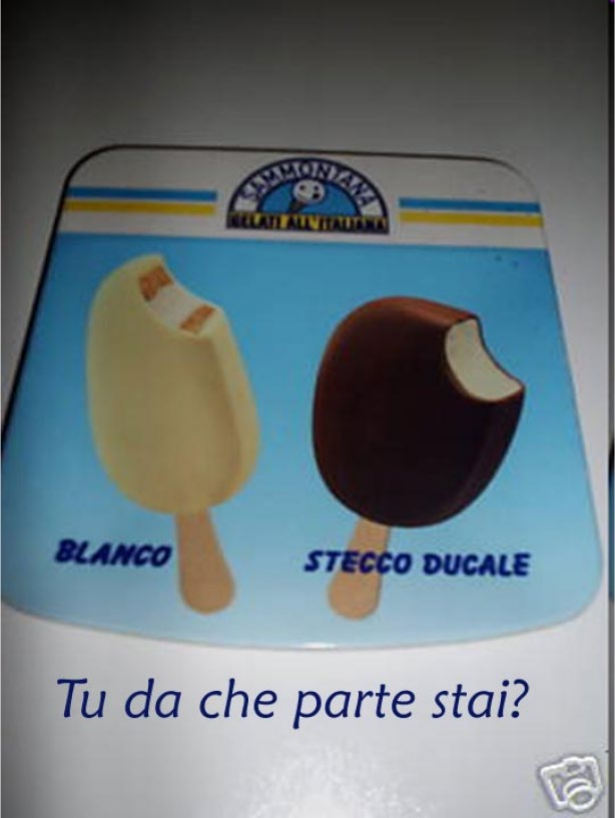
“blanco—stecco ducale Tu da che parte stai?” (blanco—stecco ducale Which side are you on?)

**Fig. 34:**
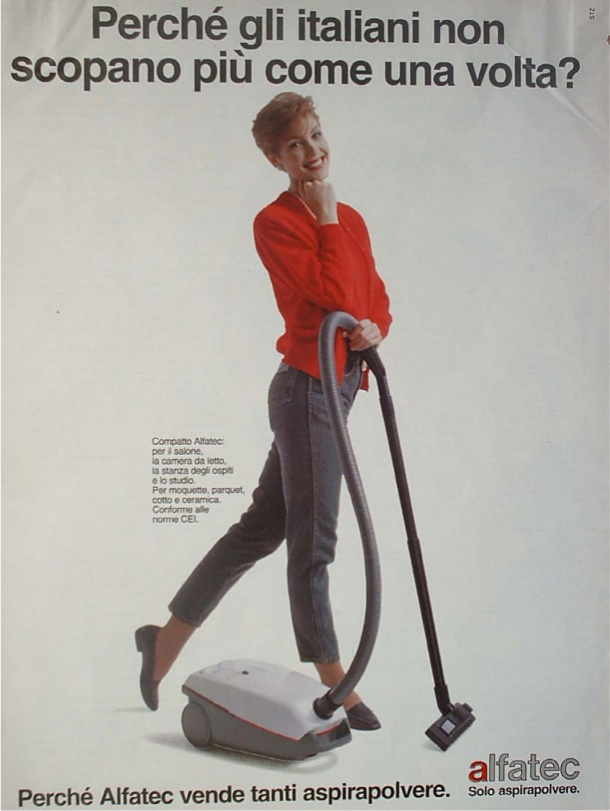
“Perché gli italiani non scopano più come una volta?—Perchè Alfatec vende tanti aspirapolvere” (Why don’t Italians sweep/fuck any more the way they used to?—Because Alfatec sells many vacuum cleaners)

In Fig. 33, the disjunctive question presupposes that you are on one of the two sides. The result is that the reader, who may previously ignore even the existence of those two icecreams, comes out of his reading the advertisement convinced that he is either on the side of Blanco or on that of Stecco Ducale, since being on no side is not an option. In Fig. 34, beside joking on the double meaning of *scopare* (‘to sweep’ and ‘to fuck’), the question *askswhy* Italians no longer do that as they once did, but *presupposesthat this is the case*. Now, once the target believes that Italians have abandoned the old ways to sweep, he will be more likely to believe that there is a cause for that, namely that Alfatec sells a lot. Which further raises the implicature that customers are satisfied with Alfatec vacuum cleaners.

**Fig. 35:**
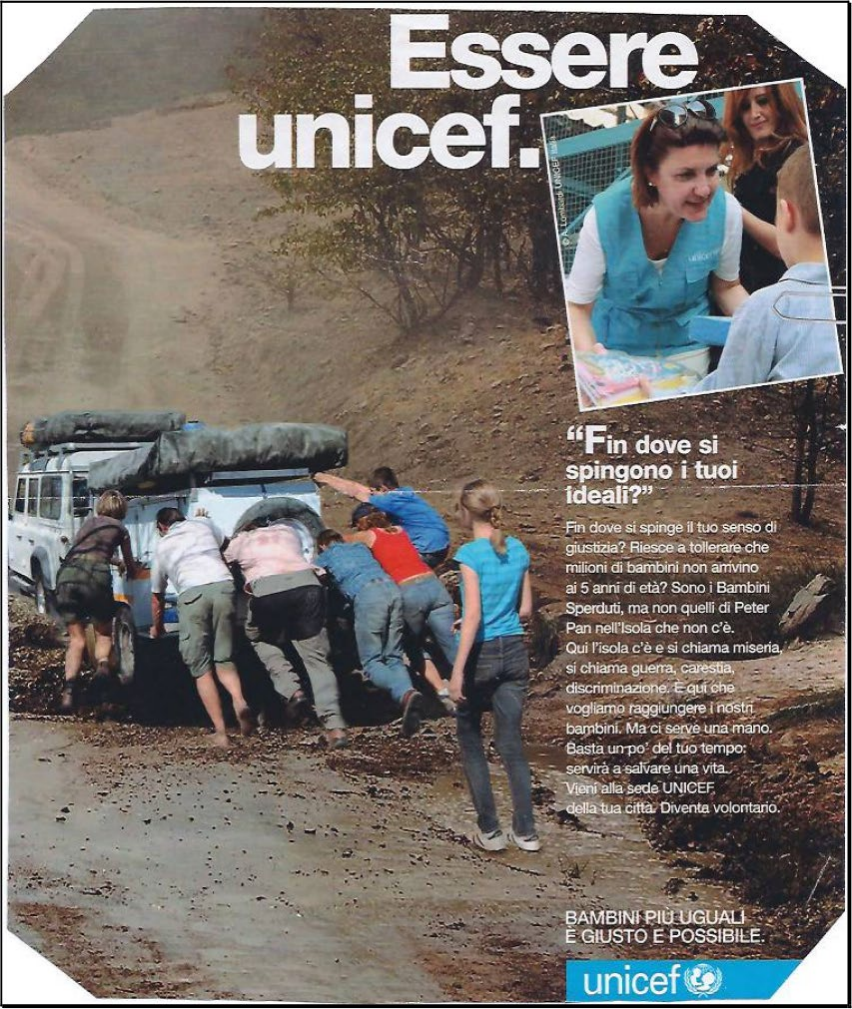
“Essere Unicef. Fin dove si spingono i tuoi ideali?” (Being Unicef. How far do your ideals arrive?)

**Fig. 36:**
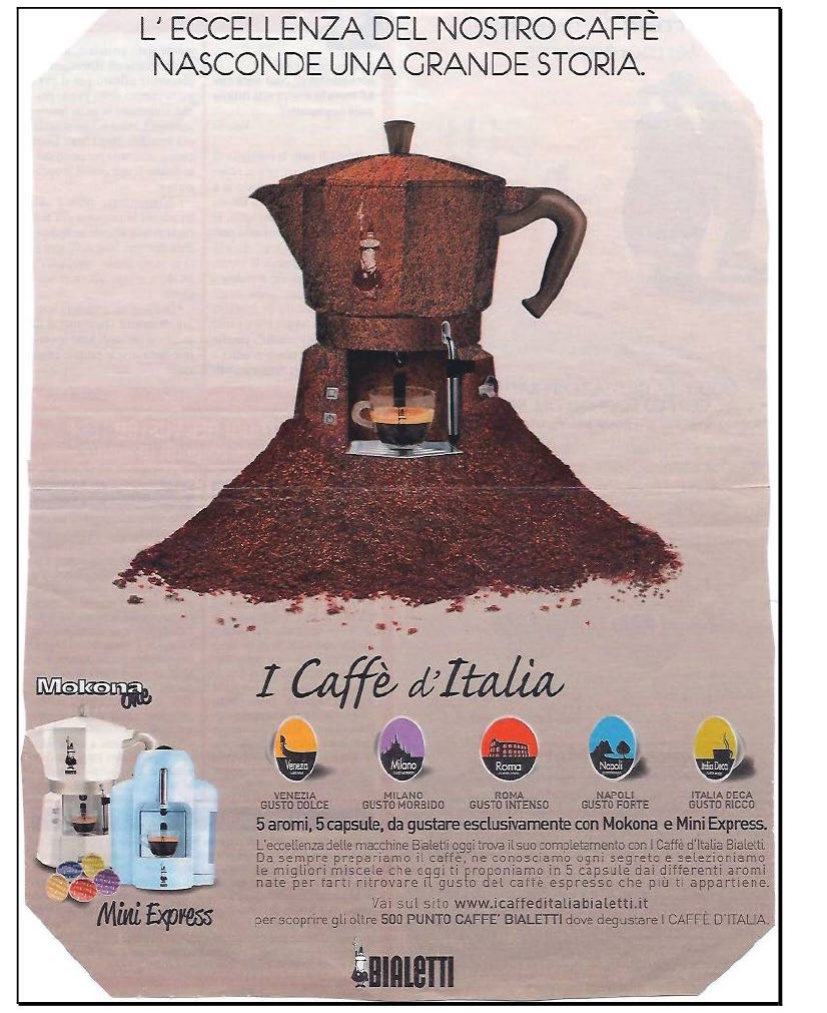
“L’eccellenza del nostro caffè nasconde una grande storia.” (The excellency of our coffee conceals a great history)

The best known activator of presuppositions, definite descriptions (cf. Strawson 1964), are frequently used in advertising to encode doubtful contents. In Fig. 35 a definite description (“your ideals”) presupposes that you have ideals. Then, a question about their progress and the juxtaposition with Unicef symbols suggest you to implicate that such ideals tend towards helping Unicef. In Fig. 36 the existence of something to be called “the excellency of our coffee” is presupposed. The assertion that this requires “a great story” is a mere pretext to utter that definite description, and the target’s being possibly not convinced about what is asserted would play no role in the success of the ad, which is completely entrusted to the presupposition.

**Fig. 37:**
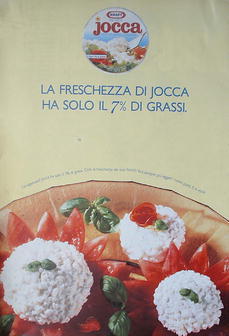
The freshness of JOCCA has only 7 % fat

**Fig. 38:**
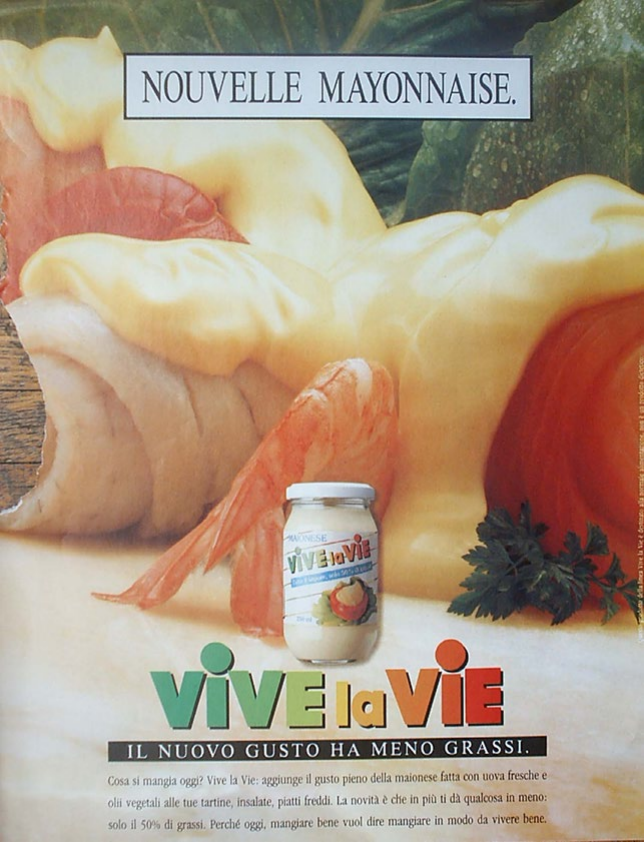
VIVE la VIE the new taste has less fat

**Fig. 39:**
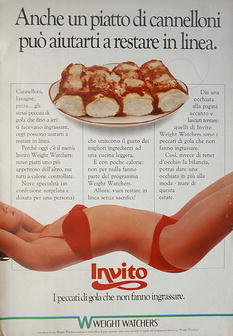
Invito. The sins of gluttony that don’t make you fat

Definite descriptions were systematically used in Italy to advertise dietetic products during the eighties. The fact that Figs. 37, 38 and 39 use exactly the same strategy is probably not casual.

It is well known that the most important feature that triggers the choice of one or the other low-fat product is the hope to find something which, beside being dietetic, also tastes good. The fact that some food belongs to the low-fat category does not need to be advertised; it is well known, and in any case not matter of persuasion: it results from the nutrition table. In other words: people decide on their own (not under the influence of ads) that they want to start a diet, and *once they are in front of the low*-*fat product shelf*, where all products are *bona fide* low-fat, they must choose which one to buy. There, the possible influence of advertisements begins. And it mainly exploits the desire to eat something pleasant in spite of the low fat content. This is exactly what the definite descriptions in the figures above do. With reference to the products, while asserting the trivial truth about their being low fat, these constructions presuppose the existence (and association to the products) of “the freshness of Jocca”, “the new taste” and “the sins of gluttony”. All of these ideas would hardly be believed by anyone if directly asserted: “Vive la Vie is The New Taste”, “Invito Weight Watchers are sins of gluttony”! But if presented as presuppositions, they don’t raise critical reactions. Then, of course, also vagueness helps. What should the Freshness of Jocca, or the New Taste mean? Such expressions have too vague a denotation to be felt as false under any respect. But, conversely, they convey very strong positive connotations.

**Fig. 40:**
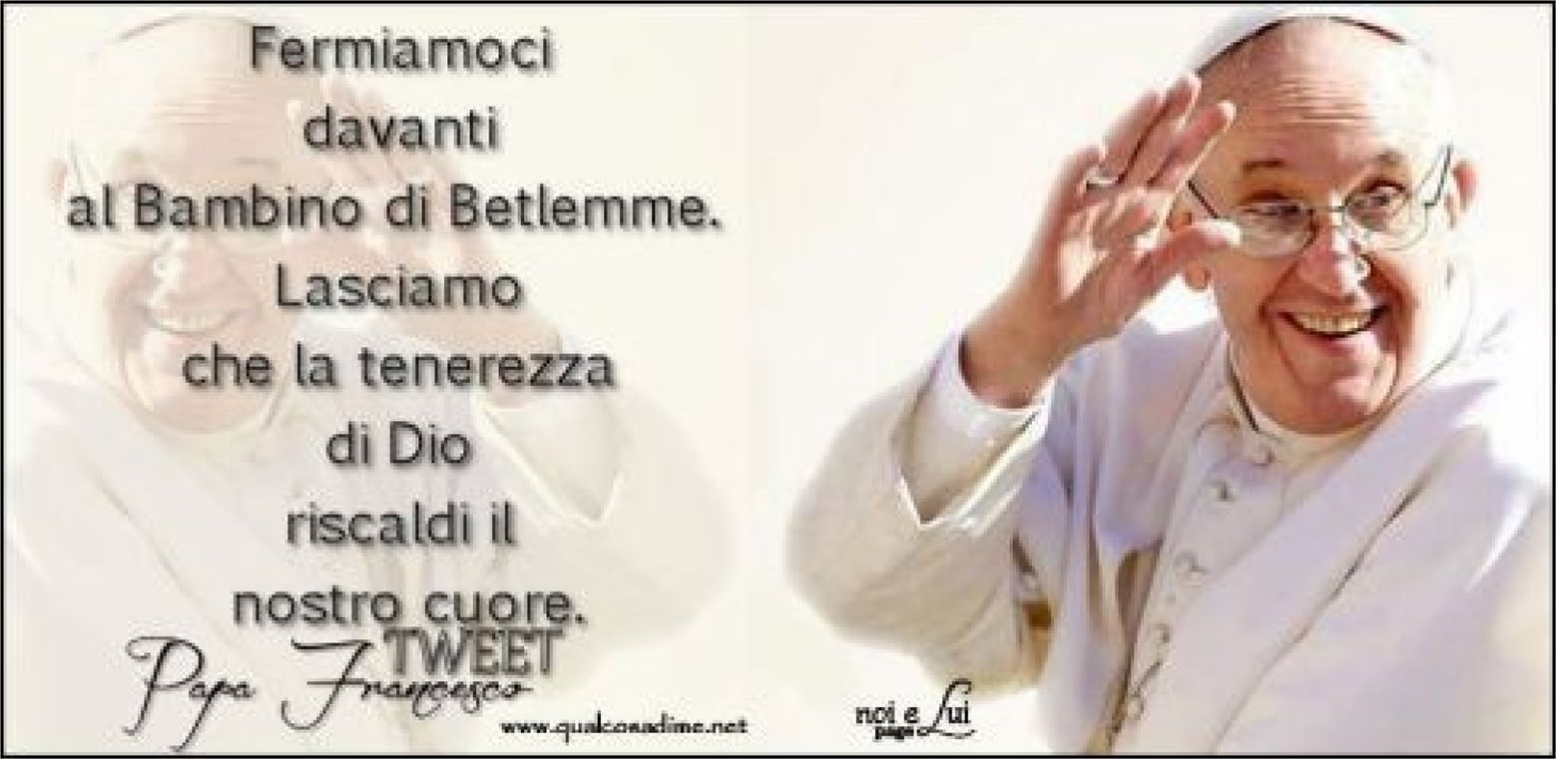
“Lasciamo che la tenerezza di Dio riscaldi il nostro cuore” (Let’s allow God’s tenderness to warm our hearts)

In Fig. 40, a quite vague and undisputable invitation to “let our hearts be warmed” is mainly the pretext to introduce by means of a definite description, and thus presuppose, the existence of God’s tenderness, which crucially includes no less than the existence of God.

**Fig. 41:**
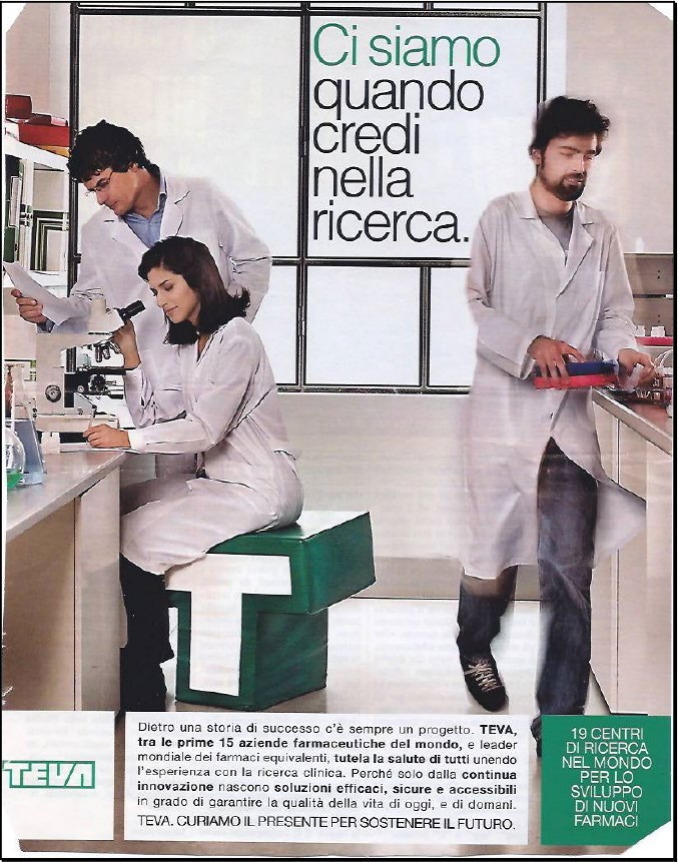
“Ci siamo/quando credi nella ricerca” (We are there/when you believe in research)

**Fig. 42:**
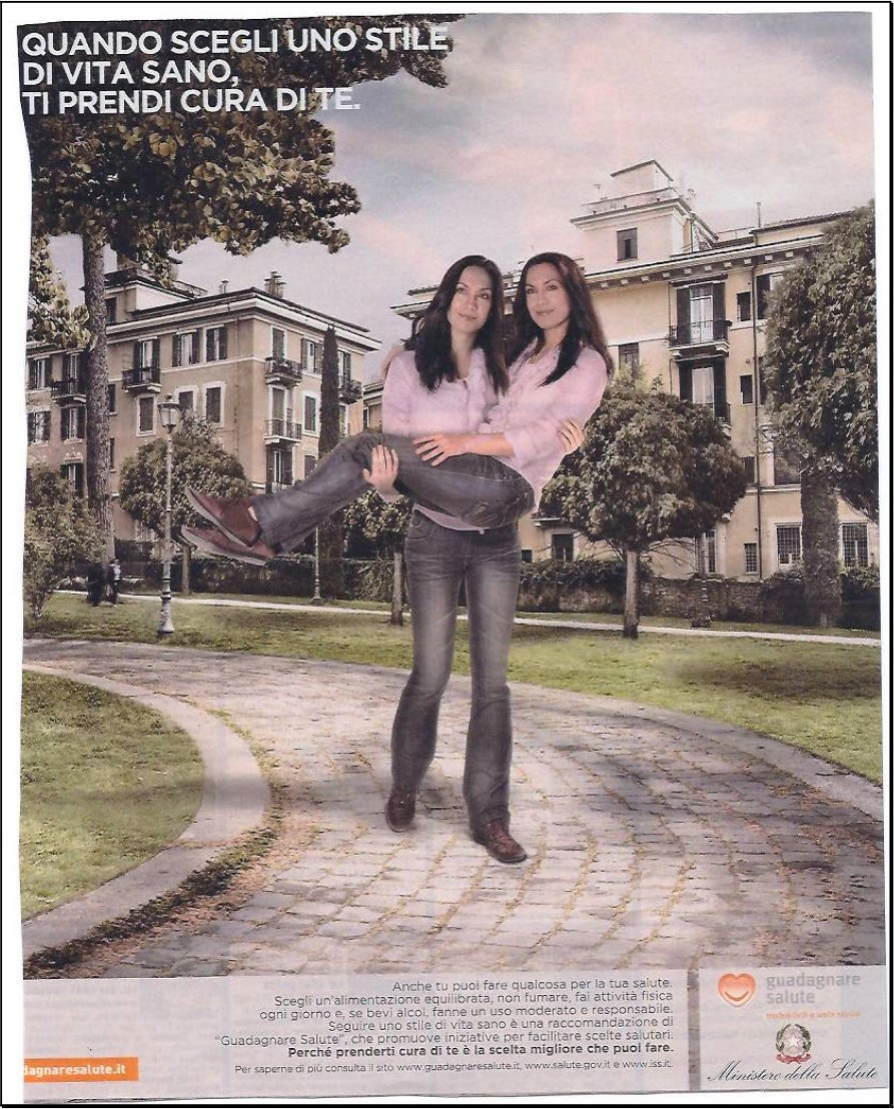
“Quando scegli uno stile di vita sano, ti prendi cura di te” (When you coose a healthy lifestyle, you take care of yourself)

In Figs. 41 and 42, temporal subordinate clauses (cf. Lombardi Vallauri 2000) presuppose respectively that you believe in research, and that you already choose a healthy lifestyle. Both are useful to let the reader feel that he is involved with the commercial proposals. The second comes with an assertion so obvious and tautological in nature, that it cannot be the aim of the message (“When you choose a healthy lifestyle, you take care of yourself”). In fact, the real purpose of the message is to convey the presupposition. In both cases, the target (who may be completely uninterested one minute before) finds himself involved in a potential situation (a *blend*, in the definition of Turner and Fauconnier 2002) which makes it more likely for him to adhere to the proposed message.

**Fig. 43:**
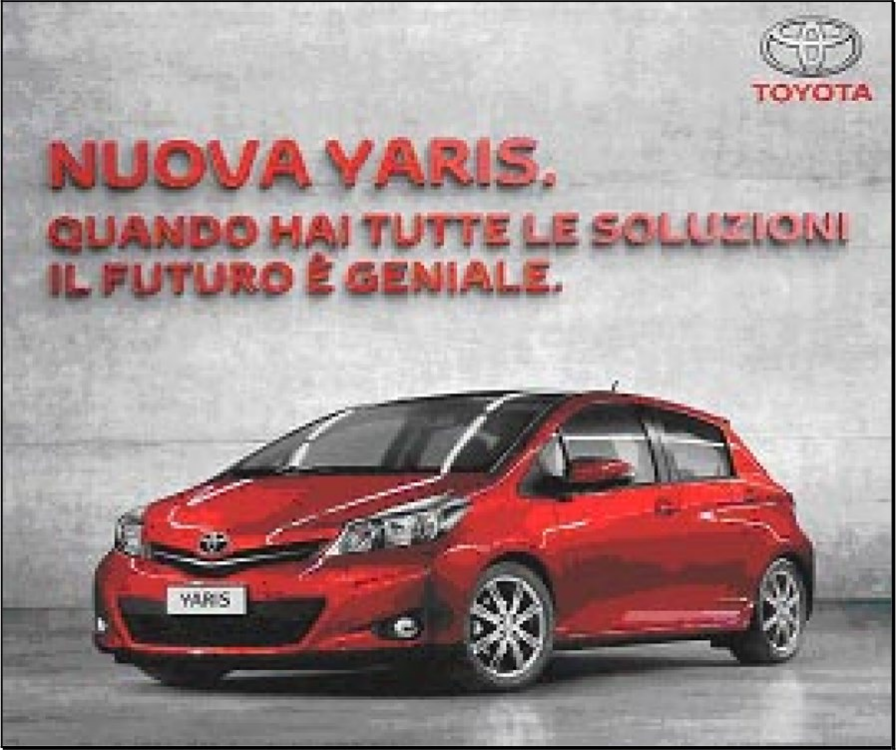
“Nuova Yaris. Quando hai tutte le soluzioni il futuro è geniale” (New Yaris. When you’ve got all solutions, the future is inspired)

In Fig. 43, the temporal subordinate clause presupposes that in some circumstances you happen to have all solutions (vague enough, but sounding desirable), and such circumstances are identified by implicature with your having a New Yaris. Moreover, Yaris is (again not explicitly, but always by means of implicature) equated to “the future”. Little importance, apart from its positive connotation, has the assertion that the future is “gifted” or “inspired”, whatever it may mean.

**Fig. 44:**
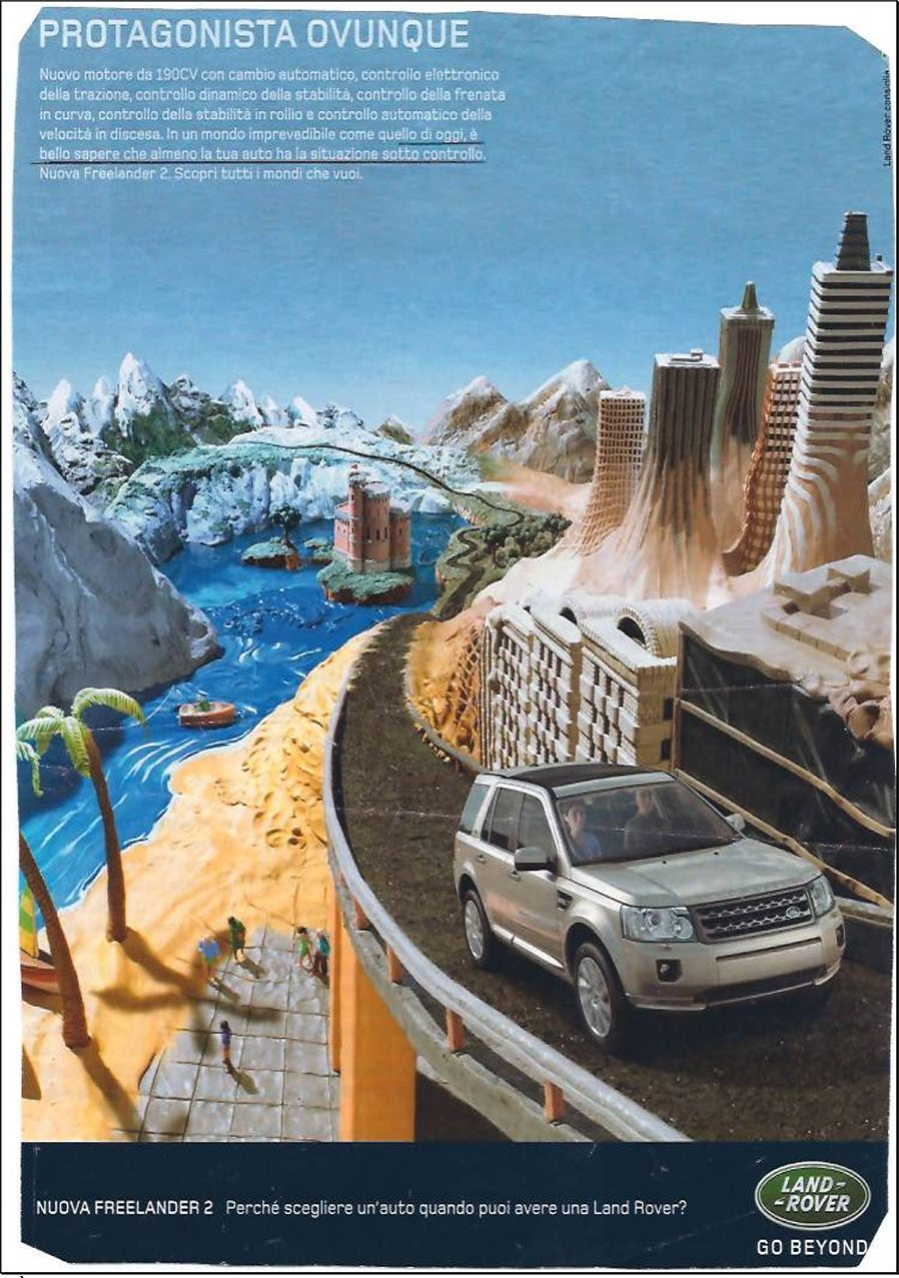
“È bello sapere che almeno la tua auto ha la situazione sotto controllo./Nuova Freelander 2. Perché scegliere un’auto quando puoi avere una Land Rover?” (“It is nice to know that at least your car has the situation under control/New Freelander 2. Why drive a car if you can drive a Land Rover?”)

In the bodycopy in Fig. 44 another typical presupposition trigger, namely the factive predicate “it is nice to know that” (Kiparsky and Kiparsky 1971) presupposes that your car has the situation under control. The juxtaposition of the text to the picture leads the reader to implicate that “your car” may be a Freelander 2. The payoff in form of a question further implicates that Land Rover Freelander is something more than a car.

### Exploitation of topics

**Fig. 45:**
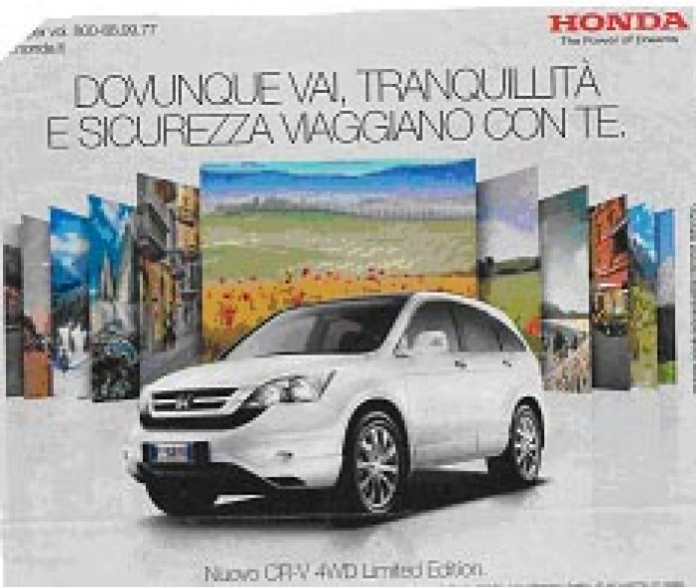
“Dovunque vai, tranquillità e sicurezza viaggiano con te” (Wherever yoy go, tranquillity and safety travel with you)

In “Topic status as a weak means of distraction” section we have pointed out that presentation as Topic can help a content to be felt as obvious, already shared, not intentionally introduced by the source, even if it is essentially new to the reader. In Fig. 45, the fact that tranquility and safety are in Topic lets the target feel them as more guaranteed, as compared to how they would appear if they were the Focus of an utterance that was organized the other way round: *Dovunque vai, viaggerai con tranquillità e sicurezza*.

**Fig. 46:**
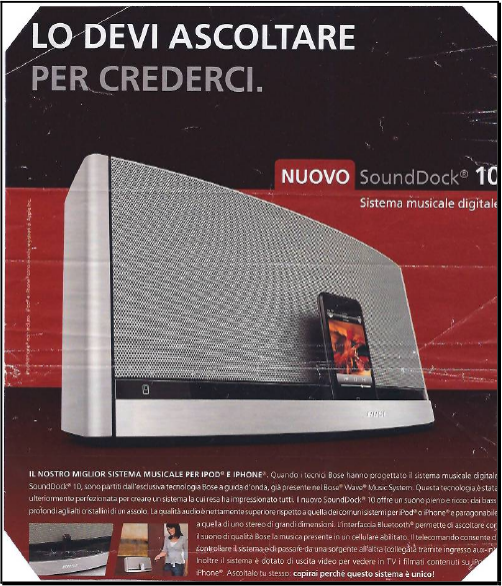
“Lo devi ascoltare/per crederci” (You must listen to it/in order to believe)

In Fig. 46, the most probable prosody of the headline is with the final purpose clause as a postposed Topic (or Appendix): *lo devi ASCOLTARE*, *per crederci*. The asserted idea that you need to listen to an audio device in order to assess its quality is trivial. On the contrary, the idea expressed as a topic, that you need to “believe” something about its sound, suggests a situation, in some way preexistent to the reading of the advertisement, in which it is hard to believe that something can produce such a sound. The same idea, if asserted, would be recognized as exaggerate; but being in Topic protects it from being questioned, in virtue of the feeling that it is widely shared.

**Fig. 47:**
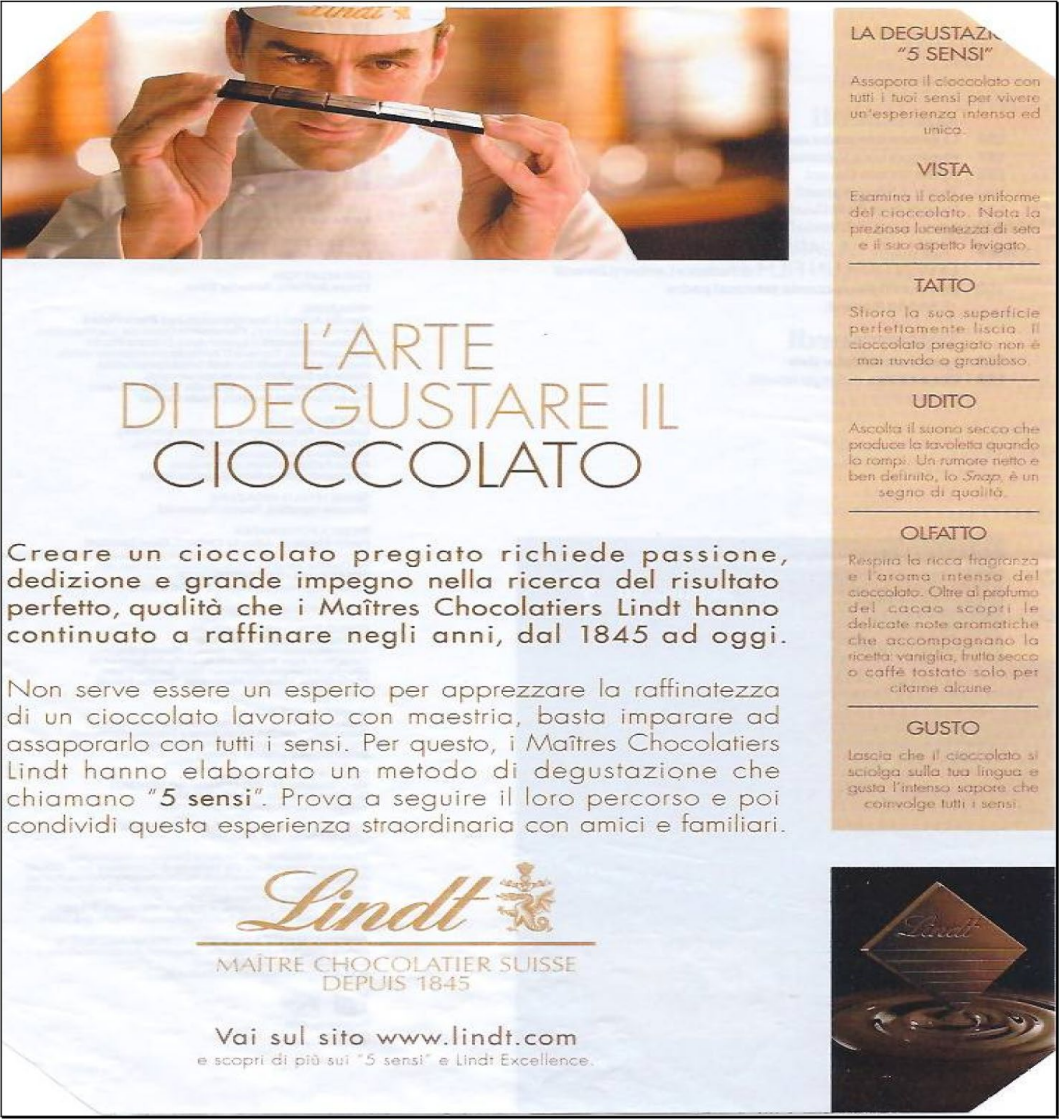
“Creare un cioccolato pregiato richiede passione, dedizione e grande impegno…” (Creating an exquisite chocolate requires passion, dedication and great diligence)

Very few people would read the whole long sentence which opens the bodycopy in Fig. 47. In fact, they will probably just read the first line. Now, the subsequent Focus (“… requires passion, dedication, diligence”) is of little use for selling the product, as compared to the initial part in Topic (“Creating an exquisite chocolate…”). Asserting “we create an exquisite chocolate” was bound to trigger more critical reactions, at least because praising oneself explicitly sounds less pleasant than acknowledging one’s success *en passant*. Moreover, the result of topicality is that the idea of Lindt’s producing exquisite chocolate is presented as obvious and widely shared. In other words, as indisputable.

**Fig. 48:**
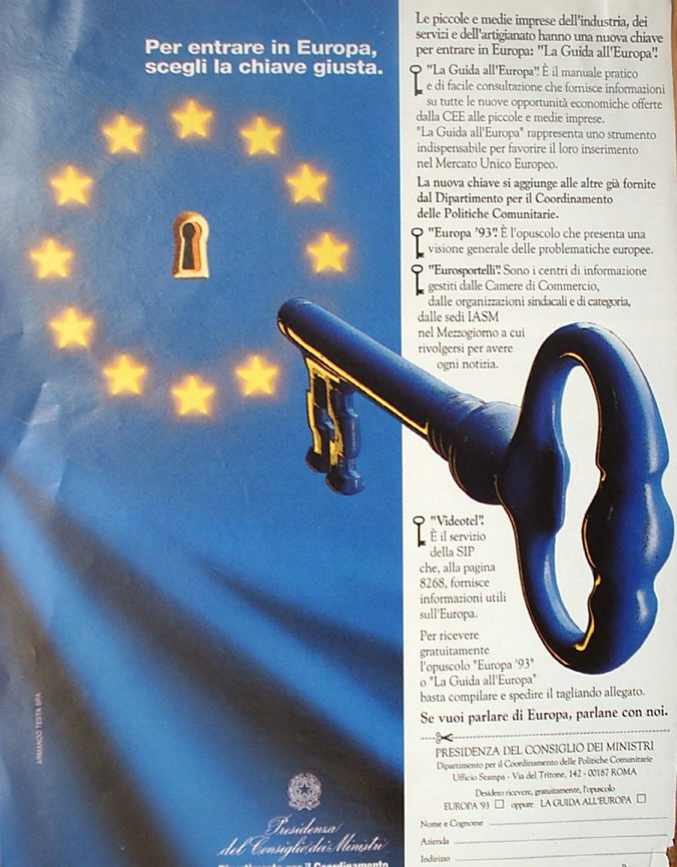
“Per entrare in Europa, scegli la chiave giusta” (To enter Europe, choose the right key

The pro-Europe advertisement by the Italian government shown in Fig. 48 presented itself as a series of instructions (“A Guide to Europe”) for Italian firms wanting to make the most of the new opportunities offered (back in the eighties) by the European economic legislation and initiatives. As it is usual in advertisements, everything written small is irrelevant as compared to the headline. Here, more precisely, all the instructions listed at the right of the page are just a *pretext* that allows to formulate the headline, where the idea that “entering Europe” is desirable is presented as already shared by means of a *topical* purpose clause. Preposed, topical purpose clauses always suggest that the aim they encode is already felt as such in the situation. This accounts for the oddity (in normal situations) of such sentences as “to dirty your shirt, you should drop chocolate or blackberry ice cream on it”. Now, presenting the desire to “enter Europe” as widely shared is precisely what this Europeanist advertisement wanted to do, in a period when many Italians were still quite skeptical.

## Conclusions

We have proposed that implicits can be divided into two fundamental categories, according to what meanings they leave not explicitly expressed in the utterance. Implicatures (cf. “Implicatures” section above) and vague expressions (cf. “Vagueness” section) belong to the *implicits of content*, in that they leave for the hearer to build part of the utterance content, having recourse to contextual cues. Presuppositions (“Presuppositions as reducers of processing effort” section) and Topics (“Topic status as a weak means of distraction” section) belong to the *implicits of responsibility*, because, while their content is explicitly encoded by actual linguistic material within the utterance, what they leave unexpressed is the act of asserting it. This very act is replaced by the allusion to some form of previous knowledge on the part of the addressee, typically encyclopedic in nature for presuppositions, and contextual in nature for Topics. Information is thus presented as already shared (in other words, as belonging already to the participants’ commitment store), and for this reason not needing that the source introduces it to the hearer.

We have further proposed that the presentation of information in implicit form can have persuasive effects. Implicatures and vague expressions partly conceal the speaker’s commitment on some content, thus possibly preventing the addressee from being aware that he is being convinced by someone else, and this because *he* is actively constructing part of the meaning of the message. Presuppositions and Topics add to this that (“The threefold function of presuppositions” section) they have extended the original function of allowing the addressee to reduce his attention on already known content: in fact, they can trigger a decrease of attention also on unknown unquestionable content, and even on questionable content, whose falseness may thus go unnoticed.

The examples we have commented on in “Some evidence from Italian commercial advertising and political propaganda” section confirm our proposal, showing that the presentation of information as implicit is a pragmatic strategy which strongly characterizes persuasive texts—and the use of such texts in relation to images—in Italian advertising, and in advertising in general. This is true both for implicits of content and implicits of responsibility. Of course their use is not limited to advertising, rather it is frequent wherever a text has persuasion as its main purpose. As a consequence, implicits conveying questionable information are usually very well represented in political speeches as well (cf. Lombardi Vallauri and Masia 2014).

## Footnotes

1 Cf. Machetti (2006).

2 Sbisà (2007: 54) further attributes this attitude to the fact that (in our translation) “Rejecting an utterance (because deemed inappropriate or not assessable as true or false) is tantamount to undermining the speaker’s authority to produce that utterance, isolating him from the communicative relationship. As the interactants in a conversation are generally likely to keep such a relationship working, they accept the utterance as appropriate and, in turn, its presuppositions.”

3 Schiffrin and Schneider (1984: 269).

4 Schneider and Chein (2003: 531).

5 To this, via implicature, it was added that their eyes would finally open if they buy a Philips television screen.

6 Whether the relevant content here is to be classified as implicated by generalized conversational implicature or presupposed, does not affect our discourse. In fact, we are precisely maintaining that similar persuasion effects can arise from implicitness of content or implicitness of responsibility.

7 More examples are described in more detail in Lombardi Vallauri (1995, 2009a, 9b, in press). Some of those commented on here, all diffused recently in Italy, already appeared in the graduation theses by Annalisa Breschi and Viviana Masia, both discussed under my guidance at Università Roma Tre.

8 We may characterize the same states of affairs in terms of the Relevance Theory; but the classical Gricean view provides a more explicit conceptual frame for the purposes of our discourse.
